# Pedohebephilia and Perceived Non-coercive Childhood Sexual Experiences: Two Non-matched Case-Control Studies

**DOI:** 10.1177/10790632221098341

**Published:** 2022-05-12

**Authors:** Sara Jahnke, Alexander F. Schmidt, Jürgen Hoyer

**Affiliations:** 1 Department of Health Promotion and Development, 9378 University of Bergen, Norway; 2 Institute of Psychology, Social & Legal Psychology, Johannes Gutenberg-Universität Mainz, Germany; 3 Institute for Clinical Psychology and Psychotherapy, 9169 Technische Universitä Dresden, Germany

**Keywords:** pedophilia, hebephilia, child sexual abuse, trauma, viewing time

## Abstract

Research on the link between childhood sexual abuse experiences (CSAE) and pedohebephilia is limited by its focus on events that the respondents rate as abusive. We asked 199 German-speaking (Study 1) and 632 English-speaking (Study 2) men with and without self-reported pedohebephilia to complete the Childhood Trauma Questionnaire (CTQ) and scales to assess perceived non-coercive childhood sexual experiences with adults (PNCSE-A), and peers (PNCSE-P, only Study 2). A substantial number of participants with PNCSE-A disagreed with all items of the CTQ Sexual Abuse subscale (e.g., 35% and 26% of pedohebephilic men in Studies 1 and 2, 38% of teleiophilic men in Study 2). While pedohebephilic men reported more CSAE than teleiophilic men, the effects for PNCSE-A did not consistently point in the expected direction. In Study 2, conviction status for sexual offenses among pedohebephilic men was linked to higher rates of CSAE, PNCSE-A, PNCSE-P, physical neglect, and physical abuse. Pedohebephilic men in Study 2 also reported more PNCSE-P than teleiophilic men. Our results highlight the importance of assessing different (positive or neutral) perceptions of CSAE. Better controlled designs (e.g., matched case-control studies) are needed to substantiate whether and how perceived non-coercive childhood sexual experiences relate to pedohebephilia and sexual offending.

Roughly 4% of men are estimated to be sexually attracted to prepubescent children (Bártová et al., 2021; Dombert et al., 2016), while about 17% report some degree of sexual attraction to pubescent children (Bártová et al., 2021). The currently predominant etiological theory of pedohebephilia (i.e., a sexual interest in prepubescent children, usually below the age of 11, and/or pubescent children, usually between 11 and 14; Seto, 2017) posits that it is caused by differences in neurodevelopment, as corroborated by markers for developmental perturbations in utero or childhood/adolescence (Fazio, 2018; Tenbergen et al., 2015). For instance, in prior research among mostly samples of men who have sexually offended, pedohebephilia has been linked to non-righthandedness, lower height, lower intelligence, and more head injuries before age 13. Other etiological models (e.g., Araji & Finkelhor, 1985) emphasize the importance of environmental factors like *childhood sexual abuse experiences* (CSAE; L. J. Cohen et al., 2010; Freund & Kuban, 1994) for the development of pedohebephilia. Yet, if we are to assume a causal role of CSAE in the development of pedohebephilia, important questions remain unexplained: Why does the great majority of CSAE victims (6% of boys and 17% of girls under 16 years in the United States; Finkelhor et al., 2014) not develop sexual attraction to children? How come most pedohebephilic men have not been sexually abused as children (Santtila et al., 2015)? And lastly, why would a young person elaborate a deeply unpleasant experience into a paraphilia?

Perhaps it is not CSAE by itself, but a combination of associated biological (e.g., neurobiological, Tenbergen et al., 2015; genetic, Alanko et al., 2016; or parental, Babchishin et al., 2019) or environmental (e.g., nonsexual adverse childhood experiences, Alanko et al., 2017; Bagley et al., 1994) factors that leads to the emergence of pedohebephilic attraction. Yet, it is also possible that some men who have been sexually abused as children do not evaluate these experiences as negative and/or may even have felt sexually aroused (Felson et al., 2019; Rind et al., 1998; Rind & Welter, 2014, 2016). Furthermore, pedohebephilic men do not only report higher rates of CSAE and non-sexual adverse childhood experiences than nonpedohebephilic men, but also higher rates of early sexual experiences with peers (Breiling et al., 2020; Santtila et al., 2010). In order to get a deeper understanding of how these different experiences might contribute to the development of a pedohebephilic attraction, the present research sought to simultaneously test the relationship between CSAE, nonsexual adverse childhood experiences, and positively perceived early sexual experiences (with peers and/or adults) and pedohebephilia. Note that we will use the term *perceived non-coercive childhood sexual experiences with adults* (PNCSE-A) to refer to participants' subjective experience of a sexual act as having been positive *and* non-coerced, not as an endorsement of adult-child sex or CSAE. Correspondingly, we will use the acronym PNCSE-P for *perceived non-coercive childhood sexual experiences with peers.*

The APA’s Board of Trustees has opted against the inclusion of a sexual attraction to pubescent children in the DSM-5 (Singy, 2015). Yet, hebephilic and pedophilic men often have similar patterns of sexual arousal to prepubescent and pubescent children (Beier et al., 2015a; Stephens et al., 2017), which, of course, are also more similar in appearance than prepubescent and post-pubescent individuals. Hence, irrespective of its nosological status, we speculate that hebephilia is not a fully distinct age sexual maturity-related erotic attraction and that hebephilia and pedophilia may have a common etiology.

## Environmental Precursors of Pedohebephilia

### Childhood Sexual Abuse Experiences

Some researchers have theorized that CSAE may lead to adult sexual attraction to children through conditioning (see Seto, 2018 for an overview). According to this view, a person would develop a sexual attraction to children as a result of sexual arousal being paired with cues of sexual activity between an adult and a child (Pfaus et al., 2001). Men appear to be more susceptible to conditioning of sexual arousal compared to women (Klucken et al., 2009), which may explain why pedohebephilia is more common in men than women (see Bártová et al., 2021 for sex differences in prevalence estimates).

While it is impossible to infer a causal role of CSAE or conditioning processes in the development of pedohebephilia based on the current state of the literature, there is ample evidence that CSAE and pedohebephilia are statistically linked. Meta-analytically combining the results of 15 studies, Jespersen et al. (2009) found a higher prevalence of CSAE among people who have sexually offended against children than among those who have offended against adults (Odds ratio = 0.51, note that an upper limit of about 50% of people who have sexually offended against children are expected to have a sexual preference for children over adults; Seto, 2018). Although most studies rely on self-reported CSAE alone, prior research has also found a link between CSAE and pedohebephilia for officially detected cases (Nunes et al., 2013). The fact that research among people who are living in the community and/or have not been convicted for sexual offenses also demonstrated a link between self-reported CSAE and pedohebephilia in clinical or community samples further attests to the robustness of the effect (Alanko et al., 2017; Bagley et al., 1994; Gerwinn et al., 2018; Grady & Levenson, 2021). Yet, as discussed in the next section, CSAE often co-occur with other potential environmental factors like nonsexual adverse childhood experiences (Alanko et al., 2017; Craissati et al., 2002; Finkelhor et al., 2007), which may confound the relationship between the two variables.

Of note, Bailey et al. (2016) found that pedohebephilic men with CSAE were more likely to have committed sexual offenses involving children than pedohebephilic community men without CSAE. Hence, CSAE might not only be linked to pedohebephilia, but also relate to a higher risk of child sexual offending among people who are sexually attracted to children.

### Nonsexual Adverse Childhood Experiences

Some theorists have proposed that attachment deficits resulting from a range of adverse childhood experiences may be the main factor driving the development of nonnormative sexual interests (Levenson et al., 2016; Ward et al., 1995). Compared to people from the general population, individuals who have sexually offended tend to report higher rates of adverse childhood experiences such as physical or emotional abuse and emotional neglect (Levenson et al., 2016). Beyond CSAE, insecure anxious or avoidant parent–child attachment, as well as emotional and physical maltreatment in the family are related to reporting any sexual interest in children among community men and women (Wurtele et al., 2014). Adults in a nationwide Finnish dataset who reported emotional maltreatment were also more likely to experience higher sexual interest in individuals below the age of 16 than study participants who did not report emotional maltreatment (Alanko et al., 2017, Study 2). An earlier study found that childhood emotional abuse (alongside other factors such as long duration and severity of CSAE) relates to increased sexual interest in and activities involving children (Bagley et al., 1994). In Gerwinn et al. (2018), nonoffending men with pedohebephilia reported more emotional neglect and higher scores on all abuse subscales of the Childhood Trauma Questionnaire (i.e., physical abuse and neglect, emotional abuse, and sexual abuse, Bernstein et al., 2003), compared to nonpedohebephilic nonoffending controls. Similar results were reported in Grady and Levenson (2021). Their online sample of men with a sexual attraction to minors were 40 times more likely to report emotional abuse and eight times more likely to report emotional neglect, compared to a sample of the male general population. Finally, Marx et al. (2020) found that self-identified pedophilic men indicated increased rates of CSAE and emotional abuse compared with nonpedophilic controls.

### Perceived Non-coercive Childhood Sexual Experiences with Adults

Individuals who report CSAE do not uniformly describe these experiences as negative (Breiling et al., 2020; Garland & Dougher, 1990; Okami, 1991; Rind et al., 1998). Compared to female victims (Trickett et al., 2011), male victims may show markedly different developmental sequelae (Rind, 2021). In a large nationally representative sample of male Finnish sixth- and ninth-graders (Felson et al., 2019), only 14% of the boys, but 51% of the girls, rated their self-reported CSAE as explicitly negative (Rind, 2022). This observation does not contradict the moral status of sex between adults and children as inacceptable (see Finkelhor, 1979; Malón, 2017 for moral arguments that do not rely on the assumption of harm).

It has been theorized from a social learning perspective that merely observing that sexual offending was pleasurable for the person perpetrating the offense might be sufficient for a victim to start showing similar behavior and to develop corresponding sexual fantasies (Burton & Meezan, 2004). Nevertheless, the distinction between different perceptions of CSA appears important, as we can expect both to be associated with different psychological (e.g., traumatization and distress vs. sexual curiosity) and behavioral sequelae (e.g., avoiding vs. approaching sexual experiences). In line with this assumption, an Israeli study among boys who have allegedly been sexually abused finds that those who reported positive reactions to the abuse (curiosity, interest, more active engagement) were more likely to engage in sexual behavior with other children later on (Hershkowitz, 2014). Yet overall, the relationship between adult sexual attraction patterns and perceived non-coercive sexual experiences with adults (PNCSE-A), such as experiences of “seduction” (Freund & Kuban, 1994), have rarely been investigated. Despite the importance that conditioning theories place on pleasurable learning experiences as a mode of reinforcement, the great majority of studies studying CSAE among pedohebephilic men focused on unwanted or coercive sexual experiences (as measured by the Childhood Trauma Questionnaire, for example, in Alanko et al., 2017).

### Perceived Non-Coercive Childhood Sexual Experiences With Peers

Some studies among community men with pedohebephilia indicate differences in the psychosexual development of pedohebephilic and teleiophilic men, particularly with respect to earlier sexual development or earlier exposure to sexual material or activities. Pedohebephilic men tend to report an earlier start of sexual maturation and activities like masturbation than teleiophilic men (Gerwinn et al., 2018; Wurtele et al., 2018). In an interview study, about half of pedohebephilic men recalled early sexual experiences with other children, ranging from “playing doctor” to penetration, before or around age 12 (Houtepen et al., 2015). Santtila et al. (2010) detected a link between sexual interest in below 16-year-olds and engagement in sexual acts with peers during participants' childhood (e.g., touching or showing one’s genitals to another child or imitating intercourse) in a sample of 1312 adult male twins (average age = 38; range 33–43). The authors interpreted this finding as in line with the conditioning theory of sexual attraction (Pfaus et al., 2001) but did not assess whether these experiences were perceived as consensual^1^ and/or pleasant for the respondents or not. Breiling et al. (2020) reported links between the presence of prepubescent sexual play or other types of sexual exploration with peers and pedophilic disorder diagnoses among men convicted for child sexual abuse. On a descriptive level, men with an exclusive pedophilic disorder reported the highest rate of prepubescent sexual play/sexual exploration with peers, followed by men with a non-exclusive pedophilic disorder and men who have not been diagnosed with pedophilic disorder. Additionally, Breiling et al. (2020) found earlier onset and higher frequencies of masturbation to be related to pedophilic attraction in people who have sexually offended against children.

## The Present Studies

To provide a more comprehensive test of environmental factors linked to sexual interests in children, we sought to assess early sexual experiences and general adverse childhood experiences among pedohebephilic and teleiophilic men. In contrast to almost the entirety of the literature on pedohebephilia, we not only assessed CSAE in general terms, but specifically asked for sexual experiences with adults that participants retrospectively rated as positive or non-coercive (both studies), as well as childhood engagement in sexual activities with peers (Study 2). In order to disentangle correlates of pedohebephilia and sexual offending, which were often confounded in prior research (Feelgood & Hoyer, 2008), we conducted two non-matched case control studies among online community samples of German-speaking (Study 1) and English-speaking (Study 2) pedohebephilic and teleiophilic men.

Our primary hypotheses are as follows: Pedohebephilic men are more likely than teleiophilic men to report (a) CSAE, (b) PNCSE-A, (c) PNCSE-P (only in Study 2), and (d) nonsexual adverse childhood experiences. Furthermore, we will explore a second set of hypotheses regarding the link between the aforementioned variables and sexual offending status of pedohebephilic men: Pedohebephilic participants with versus without prior convictions for sexual offenses report more (a) CSAE, (b) PNCSE-A, and (c) nonsexual adverse childhood experiences.

## Study 1

## Method

### Participants and Procedure

German-speaking participants were invited to our online survey via advertisements on websites, blogs, and web-forums. These were either directed at people with pedophilia or hebephilia (jungsforum.de, schicksal-und-herausforderung.de, krumme13.de, boylandonline.com, Deutsches Girlloverforum, ITP-Arcados) or science/psychology-related (e.g., forschung-erleben.uni-mannheim.de, caz-lesen.de, psychologie-heute.de, Facebook group “Psychologische Studien für alle”). We advertised the survey as a study on neurological developmental problems and traumatic childhood experiences. No compensation was offered. The research protocol was approved by the Ethics Committee at the institutional review board at Technische Universität Dresden. The survey was programmed with SosciSurvey (Leiner, 2014).

We removed 16 participants who reported to be female or who did not indicate their sex. The effective dataset contained 199 participants. In total, 89 men could be classified as teleiophilic and 101 as pedohebephilic, while nine could not be categorized into either group because they reported to be equally attracted to children and adults (see section “Self-reported sexual interests and classification of participants as pedohebephilic and teleiophilic” below for more information on the classification process). Among the pedohebephilic group, 48 were categorized as hebephilic (note that this group also includes seven participants with an equal sexual attraction to prepubescents and early-to-mid pubescents), and 53 as pedophilic. The pedohebephilic group (64% had achieved *Abitur* [i.e., university entrance certificates] as the highest school-leaving certificate in Germany) was less educated than the teleiophilic group (82% with Abitur, χ^2^ [1, *N* = 190] = 7.43, *p* = .006, φ = .198). Despite that, both groups were more educated than the average German male, as the national rate of people who have university entrance certificates is about 49% for men between age 20 and 24 (Statistisches Bundesamt, 2019). Pedohebephilic men were also about five years older than the teleiophilic men in our sample (see Table 1). Only one person from the teleiophilia group reported a sexual offense (relating to child pornography), while 29.7% of the pedohebephilic men had committed a prior sexual offense involving children (22.8% child pornography offenses, 0.0% rape, 11.9% child sexual abuse). Among the pedohebephilic men, 85.1% reported that they would have sex with a child below age 14 if it was legal and if the child gave his or her consent, while only 11.2% of the teleiophilic group agreed with this statement.

**Table 1.** Comparison of self-reported pedohebephilic and teleiophilic participants (Study 1).

Variables	α	Pedohebephilia (*n* = 101)		Teleiophilia (*n* = 89)			Mean difference 95% *CI*^b^		Mann –Whitney *U*	*d*, 95% *CI*^c^
		*M*	*SD*	*M*	*SD*	*t*(*df*)	Lower bound	Upper bound		
Self-reported attraction to prepubescents	—	7.87	2.68	1.35	0.97	22.85^***^ (128.55)	5.98	7.06	257.50^***^	3.16 [2.52, 3.91]
Self-reported attraction to early – mid pubescents	—	7.81	2.26	2.60	1.95	16.93^***^ (188)	4.57	5.83	546.00^***^	2.46 [1.95, 2.98]
Self-reported attraction to adults	—	3.87	2.50	9.54	1.02	−20.91^***^ (136.13)	−6.16	−5.13	197.50^***^	−2.91 [-3.43, −2.41]
Age	—	37.52	12.44	32.48	11.39	2.90^**^ (188)	1.54	8.52	3300.50^**^	0.42 [0.11, 0.73]
PNCSE-A	.97	1.44^a^	1.03	1.08	0.38	3.28^**^ (127.77)	0.14	0.6	3752.50^**^	0.46 [0.22, 0.65]
CTQ Sexual Abuse	.89	1.37	0.77	1.08	0.27	3.52^***^ (127.62)	0.13	0.48	3296.00^***^	0.49 [0.26, 0.65]
CTQ Emotional Neglect	.91	2.31	1.02	1.98	0.87	2.43^*^ (188)	0.06	0.64	3619.50^*^	0.35 [0.06, 0.62]
CTQ Emotional Abuse	.85	1.77	0.82	1.54	0.67	2.09^*^ (188)	0.003	0.47	3639.00^*^	0.30 [0.01, 0.57]^d^
CTQ Physical Neglect	.47	1.57	0.60	1.36	0.43	2.79^**^ (180.78)	0.07	0.38	3567.50^*^	0.40 [0.14, 0.69]
CTQ Physical Abuse	.87	1.32^a^	0.72	1.12	0.34	2.51^*^ (144.61)	0.06	0.38	3724.00^*^	0.35 [0.09, 0.54]

*Note.* Participants with an equally strong attraction to adults and prepubescents or early/mid pubescent (*n* = 9) were excluded from all analyses comparing pedohebephilic and teleiophilic participants. PNCSE-A = Perceived Non-coercive Childhood Sexual Experiences with Adults. CTQ = Childhood Trauma Questionnaire. ^*^p < .05, ^**^p < .01, ^***^p < .001

^a^*n* = 100.

^b^based on 1000 bootstrap samples, bias corrected and accelerated (BCa) intervals.

^c^bootstrap confidence intervals for Cohen’s *d* calculated with the R package bootSE based on 2000 bootstrap resamples.

^d^As these calculations are based on different bootstrap samples than the BCa intervals of the mean difference, it is possible that statistical significance changes.

## Measures

First, we assessed sexual interests utilizing both self-report and indirect latency-based viewing time (VT) measures (Schmidt et al., 2017, see Appendix for a description of procedures) followed by markers for neurodevelopmental differences (e.g., handedness, note that these results are featured in Jahnke et al., 2022), the Childhood Trauma Questionnaire (Wingenfeld et al., 2010), a measure of PNCSE-A in their own childhood, and social desirability (Ray, 1984). At the end, we collected a limited set of sociodemographic information, followed by questions about participants' willingness to have sex with children, and former criminal convictions related to child sexual abuse.

### Self-Reported Sexual Interests and Classification of Participants as Pedohebephilic and Teleiophilic

The assessment of self-reported sexual interests was based on six items from Jahnke and Malón (2019) describing male and female individuals at different stages of physical maturation (before puberty, in early-to-mid puberty, after puberty). Participants were asked to rate the degree of sexual attraction to each category (e.g., “Girls before puberty [i.e., girls who show no signs of physical maturity like pubic hair or budding breasts]") on a 10-point, Likert-type scale with response options ranging from 1 (*no sexual attraction*) to 10 (*maximum sexual attraction*). We determined relative sexual preference for children by subtracting maximum sexual attraction scores to adults from maximum sexual attraction scores to children and adolescents. Scores could range from −9 (no sexual attraction to children, maximum sexual attraction to adults) to +9 (maximum sexual attraction to children, no sexual attraction to adults).

We categorized participants based on their self-reported sexual interests as pedohebephilic (relative sexual preference for children >0, i.e., when their maximum sexual interest in children or adolescents was stronger than their maximum sexual interest in adults) or teleiophilic (relative sexual preference for children <0, i.e., when their maximum sexual interest in adults was stronger than their maximum sexual interest in children or adolescents, note that participants with a relative sexual preference for children = 0 could not be categorized into either group). The pedohebephilic group was then further subdivided into pedophilic participants (who reported a stronger sexual attraction to prepubescents compared to pubescents) and hebephilic participants (who had a stronger sexual interest in pubescent compared to prepubescent individuals or an equally strong attraction to members of both groups). While a difference of one scale point between sexual attraction to prepubescent/pubescent children and physically mature adults was enough to justify the classification of a participant as either pedohebephilic or teleiophilic, people in both groups differed considerably in their average sexual attraction to people at different stages of sexual maturity (see Table 1).

Classification based on reaction time data from the embedded VT task led to matching classifications as either pedohebephilic or teleiophilic for 127 out of 150 participants (85%), for which both indirect and direct sexual attraction scores were available (see Jahnke et al., 2022 and appendix for more information on VT procedures in the present studies). Cohen’s kappa (κ = .69) also indicated substantial agreement between the two classification procedures, according to common conventions. The point-biserial correlation between the self-report based classification and the VT difference index was *r* = .61 (*N* = 150, *p* < .001), which is noticeably higher than the agreement that recent meta-analyses find between self-reported sexual interests and VT-based scores in forensic settings (ranging between *r* = .38 and .43; Pedneault et al., 2021; Schmidt et al., 2017).

#### Childhood Trauma Questionnaire

The German short form of the Childhood Trauma Questionnaire (CTQ; Wingenfeld et al., 2010) assesses emotional abuse (e.g., “I believe that I was emotionally abused.”), physical abuse (e.g., “I was punished with a belt, a board, a cord, or some other hard object”), and sexual abuse (e.g., “Someone tried to make me do sexual things or watch sexual things”), as well as emotional neglect (e.g., " I knew there was someone to take care of me and protect me” [inversely scored]), and physical neglect (e.g., “I didn’t have enough to eat”) as a child or teenager with five statements each. All items are rated on a five-point Likert-type scale (1-*never true*, 2–*rarely true*, 3–*sometimes true*, 4–*often true,* 5-*very often true*). We calculated scale means for all abuse/neglect subscales instead of sum scores, as these allow for a more intuitive interpretation. Hence, to compare scores from this research with those reported by some other researchers (e.g., Gerwinn et al., 2018), our values have to be multiplied by five. The CTQ has an additional three-item scale to assess underreporting (Minimization/Denial) of childhood trauma. Because the utility of the CTQ Minimization/Denial subscale as a response bias index is not empirically supported (MacDonald et al., 2015), we will not analyze the corresponding results. All subscales showed internal consistencies ranging from acceptable to excellent, with the exception of the CTQ Physical Neglect scale (see Table 1).

#### Perceived Non-Coercive Childhood Sexual Experiences with Adults Scale

We developed two items to assess PNCSE-A during childhood, using a similar response format as for the CTQ. Items read: “When I was 13 years old or younger… (1) I had positive sexual experiences with an adult, (2) I engaged in sexual acts with an adult without having been coerced or forced to do so.” Note that we specified a maximum age of 13, as this corresponds with the German legal age of consent and because older children are more likely to be in later stages of puberty or to have completed puberty. Given their excellent internal consistency (see Table 1) both items were combined into one scale. It is important to note that positive and non-coerced sexual experiences are conceptually distinct, meaning that it is possible that a childhood sexual experience with an adult is experienced as coerced but positive, or as non-coerced but negative. Yet, as indicated by the high level of correlation (*r* = .94), the two concepts seem to converge in most participants’ minds or experiences in the context of adult-child sex.

#### Sociodemographic Information, Previous Convictions, and General Willingness to Engage in Sex with a Child.

We assessed participant sex (male, female, other), age, and educational achievement. Previous convictions for sexual offenses were assessed on a binary scale (yes/no) with three items (“I have been convicted for child sexual abuse,” “I have been convicted for rape,” “I have been convicted for child pornography offenses”). Participants were also asked to respond with “yes” or “no” to the item “If it was legal I would have sex with a child below 14 if the child gives his or her consent” (note that this statement is again referring to simple consent to sexual activities, which is different from informed consent as described in footnote 1).

## Planned Analyses

We dichotomized both the CTQ Sexual Abuse score and the PNCSE-A score to separate between participants who report no incidence (e.g., responding “never true” to every item) versus some degree of child sexual abuse experiences (e.g., responding something other than “never true” on at least one item). We then cross-tabulated the results to find out if there were participants who reported CSAE on only one of the two scales.

For our main analyses, we compared results for pedohebephilic and teleiophilic men, and pedohebephilic men with and without convictions for sexual offenses for each dependent variable (CTQ Sexual Abuse, Emotional Abuse, Emotional Neglect, Physical Abuse, Physical Neglect, and PNCSE-A). As childhood adverse experiences are expected to be rare in the population, we assumed that the data will contain outliers and be skewed to the right. To account for potential bias due to non-normality and the presence of outliers, we supplemented traditional *t*-tests results with two sensitivity tests: (1) bootstrapping procedures to generate bias-corrected and accelerated (BCa) confidence intervals and (2) Mann-Whitney *U*-tests. To estimate the strength of the effect, we provided Cohen’s *d* (with 95% bootstrap confidence intervals calculated with the R package bootES (Kirby & Gerlanc, 2013). Note that multiple testing increases the risks for erroneous rejection of the null hypothesis. Correcting for multiple testing, on the other hand, runs the risk of discarding potentially relevant effects (Streiner, 2015). Therefore, the present study did not correct for multiple testing. While we did not conduct a priori power analyses, a posteriori power analyses indicated that *t*-tests could detect effect sizes as small as *d* = 0*.*20 with α = .05, 1 – β = .80 with a sample of 190 participants (counting only participants who could be classified as teleiophilic or pedohebephilic).

## Results

### Descriptive Results

A distribution of responses to each item of the PNCSE-A is provided in Supplemental Material C, Table S7. We assessed the percentage of those who responded with anything other than “never” to any item of the CTQ Sexual Abuse subscale or PNCSE-A. On a descriptive level, pedohebephilic men were about three times as likely to report any CTQ Sexual Abuse (39% or 39 out of 101) than teleiophilic men (13% or 12 out of 89). Note that 9.3% of German men in a representative study reported any degree of CSAE on the CTQ Sexual Abuse subscale (Witt et al., 2017). Pedohebephilic men were also about four times as likely to report any PNCSE-A (20% or 20 out of 100) compared to teleiophilic men (5% or four out of 89). Nevertheless, more than half of our pedohebephilic participants (54%) and the large majority of our teleiophilic participants (85%) fully denied any involvement in sexual acts with adults while they were young, irrespective of whether these experiences were rated as positive/consensual and/or abusive. Among the pedohebephilic sample, 20% reported PNCSE-A. Strikingly, about a third of the pedohebephilic men who indicated PNCSE-A completely denied CSAE on all items of the CTQ Sexual Abuse items (seven out of 20, in absolute values). Among the four teleiophilic men who reported PNCSE-A, one denied having been sexually abused on all items of the CTQ Sexual Abuse subscale.

### Comparison of Pedohebephilic and Teleiophilic Men

Pedohebephilic participants were significantly more likely to report PNCSE-A and scored higher on every CTQ Abuse/Neglect subscale (see Table 1 for *t*-test results, BCa 95% *CI*s, and bootstrap estimates of Cohen’s *d*). Effect sizes in the present study ranged from *d* = 0.30 to *d* = 0.49, indicating conventionally small effect sizes (J. Cohen, 1992). The results of non-parametric tests (Mann-Whitney *U*-test), which are also included in Table 1, are in line with those obtained via parametric tests. Correlational analyses between all variables showed conventionally small to large intercorrelations between PNCSE-A on one side and age and the five CTQ subscales on the other side (see Table 2). Relative sexual preference for children remained positively related to all traumatic and nontraumatic childhood experiences when controlling for age as a potential confound [partial correlations are displayed in Table 2, above the diagonal]). For exploratory comparisons between pedophilic, hebephilic, and teleiophilic men, see Table S1 in the Supplemental Material A.

**Table 2.** Intercorrelations among study variables (Study 1, *N* = 199).

Variables	PNCSE-A	CTQ SA	CTQ EN	CTQ EA	CTQ PN	CTQ PA	Age	RSPC
PNCSE-A	—	.37^***^	.10	.11	.07	.06	—	.25^***^
CTQ Sexual Abuse (SA)	.39^***^	—	.24^***^	.33^***^	.23^**^	.46^***^	—	.22^**^
CTQ Emotional Neglect (EN)	.16^*^	.28^***^	—	.55^***^	.64^***^	.37^***^	—	.14
CTQ Emotional Abuse (EA)	.11	.33^***^	.53^***^	—	.39^***^	.51^***^	—	.14
CTQ Physical Neglect (PN)	.10	.25^***^	.66^***^	.39^***^	—	.41^***^	—	.13
CTQ Physical Abuse (PA)	.09	.48^***^	.41^***^	.51^***^	.43^***^	—	—	.12
Age	.18^*^	.16^*^	.35^***^	.04	.20^**^	.19^**^	—	—
RSPC	.28^***^	.25^***^	.19^**^	.14^*^	.17^*^	.15^*^	.20^*^	—

*Note.* Variables below the diagonal are bivariate correlations, variables above the diagonal are partial correlations (controlling for the effect of age). PNCSE-A = Perceived Non-coercive Childhood Sexual Experiences with Adults. CTQ = Childhood Trauma Questionnaire. RSPC = Relative Sexual Preference for Children (i.e., self-reported maximum sexual attraction to prepubescent and early/mid pubescent children - self-reported maximum sexual attraction to mature adults). ^*^p < .05, ^**^p < .01, ^***^p < .001.

### Comparison of Pedohebephilic Men With and Without Convictions for Sexual Offenses

Besides the pedohebephilic group with convictions for sexual offenses being more than 10 years older, we found no significant differences between pedohebephilic men who have and have not been convicted for sexual offenses (see Table 3, but note that the sample size is likely to be too small to detect significant effects). The findings were confirmed in two sensitivity tests (based on BCa confidence intervals and Mann-Whitney *U*-tests, see Table 3). Descriptively, effects are pointing in the hypothesized direction of people with convictions reporting higher rates of physical abuse and emotional neglect (all *ns.*, and |*d*s| ∼ 0.30), as well as a marginally stronger relative sexual preference for children (|*d*| = 0.11, *ns.*). Contrary to expectations, men with convictions for sexual offenses reported marginally lower rates of CTQ Sexual Abuse and PNCSE-A (all *ns.* and |*d*s| ∼ 0.10).

**Table 3.** Comparison of self-reported pedohebephilic participants with and without convictions for sexual offending (Study 1).

Variables	No history of sexual offenses (*n* = 71)		History of sexual offenses (*n* = 30)			Mean difference 95% *CI*^a^		Mann-Whitney *U*	*d*, 95% *CI*^d^
	*M*	*SD*	*M*	*SD*	*t*(*df*)	Lower bound	Upper bound		
RSPC	5.58	2.59	5.87	2.75	−0.50 (99)	−1.55	1.02	982.50	−0.11 [-0.56, 0.34]
Age	34.03	10.09	45.80	13.69	−4.25^***^ (42.90)	−17.67	−6.48	524.50^***^	−1.05 [-1.56, −0.52]
PNCSE-A	1.47^b^	1.02	1.37	1.06	0.47 (98)	−0.41	0.53	998.50	0.10 [-0.35, 0.50]
CTQ Sexual Abuse	1.40	0.87	1.30	0.48	0.58 (99)	−0.17	0.34	956.50	0.13 [-0.25, 0.41]
CTQ Emotional Neglect	2.21	0.96	2.55	1.12	−1.54 (99)	−0.81	0.13	885.00	−0.34 [-0.77, 0.12]
CTQ Emotional Abuse	1.79	0.88	1.75	0.69	0.22 (99)	−0.29	0.37	997.50	0.05 [-0.37, 0.41]
CTQ Physical Neglect	1.58	0.57	1.55	0.66	0.18 (99)	−0.30	0.30	994.00	0.04 [-0.41, 0.47]
CTQ Physical Abuse	1.26	0.62	1.47^c^	0.91	−1.29 (98)	−0.65	0.14	857.50	−0.28 [-0.74, 0.20]

RSPC = Relative Sexual Preference for Children (i.e., self-reported maximum sexual attraction to prepubescent and early/mid pubescent children - self-reported maximum sexual attraction to mature adults). PNCSE-A = Perceived Non-coercive Childhood Sexual Experiences with Adults. CTQ = Childhood Trauma Questionnaire. ^*^p < .05, ^**^p < .01, ^***^p < .001.

^a^= based on 1000 bootstrap samples, bias corrected and accelerated (BCa) intervals.

^b^*n* = 70.

^c^*n* = 29.

^d^bootstrap confidence intervals for Cohen’s *d* calculated with the R package bootSE based on 2000 bootstrap resamples.

### Alternative Classification Procedure

While the two methods of classifying participants as pedohebephilic or teleiophilic (VT and self-report) showed substantial agreement, results of the VT task, when available, did not always agree with self-reports. We focused our previous analyses on self-reports, which we believed to be reliable in an anonymous, non-forensic setting. Nevertheless, to assess the robustness of the results based on self-report, we re-conducted the analyses using only those participants who had agreement on the implicit and explicit classification procedures (*N* = 127). Findings are presented in Supplemental Material B (Table S3 for group comparisons, Table S4 for correlational analyses). Despite reduced analytic power, we again found that the teleiophilic sample was significantly less likely to report PNCSE-A, sexual abuse, emotional neglect and abuse and physical neglect than pedohebephilic men. Although the effect sizes were highly similar (*d* = 0.35 for self-report based classifications, *d* = 0.37 for concordant classification based on self-report and VT), differences between the groups on CTQ Physical Abuse did not reach significance in the test based on only those with concordant self-report and VT measures (with the exception of BCa 95% *CI*s). Hence, we were able to corroborate our previous test strategy based on self-reports with a substantially more conservative classification and test procedure.

## Study 2

## Method

### Participants and Procedure

To recruit sufficient numbers of participants with pedohebephilic and teleiophilic interests, data were collected via B4U-ACT and MTurk. B4U-ACT (www.b4uact.org) is an advocacy group for minor-attracted people, which posted the link to the survey in their support group between July, 2018 and March, 2019. Participation was incentivized by offering a donation of US$1.50 to B4U-ACT for each participant (with a maximum sum of US$300). Mturk is a marketplace for recruiting workers to conduct any online tasks that require human intelligence, which has gained popularity as a means to recruit participants for online surveys (Buhrmester et al., 2011). Mturk workers were eligible for participation if they had been approved for 100 to 5000 human intelligence tasks with an overall approval rate of at least 80%. They received US$2.00 for participation. While the survey language was English, we accepted Mturk workers from Australia, Austria, Belgium, Canada, Czech Republic, Denmark, Estonia, Finland, France, Germany, Greece, Iceland, Italy, Lithuania, Luxembourg, Netherlands, New Zealand, Norway, Poland, Portugal, Slovakia, Spain, Sweden, United Kingdom, and the United States. The study was advertised as a survey on “wanted and unwanted childhood sexual experiences, cognitive development, and sexual interests in children or adults among men from the community”. As in Study 1, we used SosciSurvey (Leiner, 2014) to collect data. In total, we recruited 329 participants on B4U-ACT and 320 on Mturk. 17 people were excluded because they skipped the question about their sex or stated “female” or “other”. Hence, the final sample included 632 participants.

We categorized 141 men as pedophilic, 137 as hebephilic, and 317 as teleiophilic. Thirty-seven participants reported an equally strong pedohebephilic and teleiophilic attraction and therefore could not be classified as teleiophilic or pedohebephilic. Thirty-one participants in the hebephilic group reported an equally strong sexual attraction to prepubescent and pubescent children.

In the pedohebephilic group, 53% had achieved an Associate degree, BA degree or higher, compared to 62% in the teleiophilic group (χ^2^ [1, *N* = 593] = 5.58, *p* = .018, φ = 10). There was no significant group difference with respect to age (see Table 4). Among the pedohebephilic group, 14% reported convictions for sexual offenses (1.4% rape, 6.8% child sexual abuse, 10.1% child pornography offenses), while only 1.3% of the teleiophilic men reported previous convictions for sexual offenses (0.0% rape, 0.9% child sexual abuse, 0.3% child pornography offenses). In the pedohebephilic group, 86% (compared to 7% in the teleiophilic group) agreed that if it was legal, they would have sex with a child below 14 if the child gives his or her consent.

**Table 4.** Comparison of self-reported pedohebephilic and teleiophilic participants (Study 2).

Variables	α	Pedohebephilia (*n* = 278)		Teleiophilia (*n* = 317)			Mean difference 95% *CI*^b^		Mann-Whitney *U*	*d*, 95% *CI*^e^
		*M*	*SD*	*M*	*SD*	*t*(*df*)	Lower bound	Upper bound		
Self-reported attraction to prepubescents	—	8.23	2.36	1.30	1.09	45.04^***^ (379.21)	6.66	7.22	1213.50^***^	3.86 [3.37, 4.39]
Self-reported attraction to early – mid pubescents	—	7.99	2.38	1.78	1.71	36.06^***^ (495.35)	5.89	6.56	3619.00^***^	3.03 [2.63, 3.42]
Self-reported attraction to adults	—	4.44	2.61	9.62	0.91	−31.53^***^ (335.76)	−5.48	−4.87	1771.00^***^	−2.73 [−2.97, −2.48]
Age	—	34.44^c^	12.98	35.23	11.14	−0.79 (539.54)	−2.71	0.94	39,422.00	−0.07 [−0.23, 0.10]
PNCSE-A	.88	1.50	1.09	1.42^a^	0.99	0.93 (592)	−0.10	0.28	42,951.00	0.08 [−0.08, 0.23]
PNCSE-P	.94	2.61	1.56	1.88^a^	1.26	6.25^***^ (531.22)	0.48	0.99	31878.00^***^	0.52 [0.35, 0.69]
CTQ Sexual Abuse	.92	1.58	1.02	1.30^a^	0.71	3.88^***^ (485.95)	0.14	0.43	35497.50^***^	0.33 [0.18, 0.48]
CTQ Emotional Neglect	.89	2.33	0.96	2.16^a^	0.93	2.15^*^ (592)	0.02	0.35	39382.00^*^	0.18 [0.01, 0.34]
CTQ Emotional Abuse	.87	1.89^d^	0.90	1.82^a^	0.90	1.03 (591)	−0.06	0.23	40,411.50	0.09 [−0.07, 0.25]
CTQ Physical Neglect	.73	1.47	0.60	1.58^a^	0.66	−2.03^*^ (592)	−0.20	0.005	39858.50^*^	−0.17 [−0.33, 0.01]
CTQ Physical Abuse	.81	1.39	0.59	1.51	0.72	−2.29^*^ (588.92)	−0.23	−0.02	40,200.50	−0.19 [−0.35, −0.04]

*Note.* Participants with an equally strong attraction to adults and prepubescents or early/mid pubescents were excluded from pubescent (*n* = 37) were excluded from all analyses comparing pedohebephilic and teleiophilic participants. PNCSE-A = Perceived Non-coercive Childhood Sexual Experiences with Adults. PNCSE-P = Perceived Non-coercive Childhood Sexual Experiences with Peers. CTQ = Childhood Trauma Questionnaire. ^*^p < .05, ^**^p < .01, ^***^p < .001.

^a^*n* = 316.

^b^based on 1000 bootstrap samples, bias corrected and accelerated (BCa) intervals.

^c^*n* = 273.

^d^*n* = 277.

^e^bootstrap confidence intervals for Cohen’s *d* calculated with the R package bootSE based on 2000 bootstrap resamples.

Sample size was a priori planned to detect differences as small as *d* = 0.21 for reasons unrelated to the current research (Jahnke et al., 2022), using G*Power (Faul et al., 2007). Hence, the current survey is sufficiently powered for the planned comparisons of pedohebephilic and teleiophilic men on sexual abuse histories for small effects down to *d* = 0.31, as detected in Study 1 (1 - β = .98). The study was greenlighted by the university ethics board of the MSH Medical School Hamburg, Germany, where the second author was employed during the planning and launch phase of this survey.

### Measures

The scales were presented in the same order in that they are listed below. Note that we additionally assessed markers for neurodevelopmental differences (e.g., IQ), which are featured in a separate publication (Jahnke et al., 2022).

#### Self-Reported Sexual Interests and Classification of Participants as Pedohebephilic or Teleiophilic

We used the English version of the scale from Study 1, and classified participants following the same procedures. The VT measures corroborated the validity of the self-reports for 381 (77%) of those participants, for which direct and indirect measures of sexual maturity interests were available (*n* = 493). That means that the great majority of participants was categorized as teleiophilic or pedohebephilic according to both procedures (see Jahnke et al., 2022 and the Appendix for more information on the VT measure). This indicates a moderate agreement according to common standards (Cohen’s κ = .50). The point-biserial correlation between the self-report based classification and the VT difference index was *r* = .50, again surpassing the agreement between self-report and VT-based scores in forensic samples (Pedneault et al., 2021; Schmidt et al., 2017).

#### Childhood Trauma Questionnaire

We used the English original version of the CTQ scales employed in Study 1 (Bernstein et al., 2003). In Study 2, internal consistencies were acceptable for all subscales (see Table 4).

#### Perceived Non-Coercive Childhood Sexual Experiences with Adults and Peers Scales

We used the two items from Study 1 to assess PNCSE-A. Additionally, we added two similar items to tap into corresponding sexual experiences with peers (PNCSE-P; “I had positive sexual experiences with same-aged peers (not older or younger than 2 years of age compared to myself)” and “I engaged in sexual acts with same-aged peers (not older or younger than 2 years of age compared to myself) without having been coerced or forced to do so.”). We averaged the results of these two peer-related items to create the PNCSE-P score. Cronbach’s α indicated excellent internal consistency for both scales (Table 4).

#### Sociodemographic Information, Previous Convictions, and General Willingness to Engage in Sex with a Child

We used the same items as in Study 1.

### Planned Analyses

See Study 1 (adding PNCSE-P as an additional factor in the analyses).

## Results

### Descriptive Results

A distribution of responses to each item of the PNCSE-A and PNCSE-P is provided in Supplemental Material C, Table S7. We assessed the percentage of those who responded with anything other than “never” to any item of the CTQ Sexual Abuse subscale, PNCSE-A, or PNCSE-P. On a descriptive level, 44% (122 out of 278) of the pedohebephilic men and 25% (78 out of 316) of the teleiophilic men reported any instances of CSAE on the respective CTQ subscale. Twenty-two percent (62 out of 278) of the pedohebephilic and 21% (65 out of 316) of the teleiophilic group self-reported PNCSE-A. When both scales were taken together, 33% (103 out of 316) of teleiophilic men and 50% (138 out of 278) of pedohebephilic men reported CSAE, irrespective of whether they themselves perceived these experiences as abusive, positive/consensual, or both. Among the pedohebephilic men, 26% (or 16 out of 62) of those who indicated PNCSE-A did not endorse any sexual abuse on the respective CTQ subscale (38% or 25 out of 65 in the teleiophilic group). A significant proportion of both groups recalled PNCSE-P (64% [177 out of 278] and 41% [131 out of 316] in the pedohebephilic and the teleiophilic group, respectively).

### Comparison of Pedohebephilic and Teleiophilic Men

Again, we conducted *t*-tests to compare test scores in the pedohebephilic and the teleiophilic group. In contrast to Study 1 (*d* = 0.46), results from Study 2 did not show that pedohebephilic men reported more PNCSE-A during their childhood (*d* = 0.08). Yet, pedohebephilic men were more likely than teleiophilic men to report PNCSE-P (*d* = 0.52; see Table 4 for *t*-test results, BCa 95% *CI*s, and bootstrap estimates for Cohen’s *d*). As in Study 1, we found that effect sizes were generally small for differences between pedohebephilic and teleiophilic men on the four nonsexual CTQ subscales (Table 4). However, note that some of these effects in Study 2 are in the opposite direction of the effects in Study 1. Specifically, for CTQ Physical Abuse and CTQ Physical Neglect, pedohebephilic men were *less* likely to have experienced these types of maltreatment than teleiophilic men (*d* = −0.17 for physical abuse, *d* = −0.19 for physical neglect), while the effects were in the expected positive direction for CTQ Emotional Abuse (*d* = 0.09) and CTQ Emotional Neglect (*d* = 0.18). Overall, sensitivity tests corroborated the findings based on conventional *t*-tests, with the exception of CTQ Physical Neglect (95% BCa *ns*.) and CTQ Physical Abuse (Mann-Whitney Test *ns*.).

Correlational analyses indicated small to medium-sized associations between PNCSE-A, CTQ Sexual Abuse, and PNCSE-P (Table 5). Self-reported PNCSE-P was also positively related to age. The relative sexual preference for children was (weakly) related only to PNCSE-P, CTQ Sexual Abuse, CTQ Emotional Neglect, and CTQ Physical Neglect (note that all correlations were positive with the exception of the link to CTQ Physical Neglect). Exploratory comparisons between pedophilic, hebephilic, and teleiophilic men are presented in Table S2 in the Supplemental Material A.

**Table 5.** Intercorrelations of study variables (Study 2, *N* = 625–631).

Variables	PNCSE-A	PNCSE-P	CTQ SA	CTQ EN	CTQ EA	CTQ PN	CTQ PA	Age
PNCSE-A	—							—
PNCSE-P	.46^***^	—						—
CTQ Sexual Abuse (SA)	.46^***^	.37^***^	—					—
CTQ Emotional Neglect (EN)	.12^**^	.13^**^	.31^***^	—				—
CTQ Emotional Abuse (EA)	.19^***^	.18^***^	.46^***^	.61^***^	—			—
CTQ Physical Neglect (PN)	.34^***^	.15^***^	.45^***^	.54^***^	.60^***^	—		—
CTQ Physical Abuse (PA)	.29^***^	.16^***^	.43^***^	.41^***^	.67^***^	.54^***^	—	—
Age	.02	.11^**^	.06	.05	.04	-.05	.04	—
RSPC	.05	.26^***^	.17^***^	.11^**^	.05	-.08^*^	-.07	.00

*Note.* PNCSE-A = Perceived Non-coercive Childhood Sexual Experiences with Adults. PNCSE-P = Perceived Non-coercive Childhood Sexual Experiences with Peers. CTQ = Childhood Trauma Questionnaire. RSPC = Relative Sexual Preference for Children (i.e., self-reported maximum sexual attraction to prepubescent and early/mid pubescent children - self-reported maximum sexual attraction to mature adults). ^*^p < .05, ^**^p < .01, ^***^p < .001.

### Comparison of Pedohebephilic Men with and Without Convictions for Sexual Offenses

We found that pedohebephilic men who reported prior convictions indicated a significantly higher relative sexual preference for children, higher age, and more PNCSE-A, PNCSE-P, CTQ Sexual Abuse, CTQ Physical Neglect, and CTQ Physical Abuse compared to pedohebephilic men without prior convictions (see Table 6). The findings were robust across two sensitivity tests (95% BCa *CI*, Mann-Whitney *U*-test). With effect sizes between |*d*| = 0.46 and |*d*| = 0.57, effects can be classified as small to medium, according to Cohen’s (1992) conventions.

**Table 6.** Comparison of self-reported pedohebephilic participants with and without convictions for sexual offending (Study 2).

Variables	No history of sexual offenses (*n* = 239)		History of sexual offenses (*n* = 39)			Mean difference 95% *CI*^a^		Mann-Whitney *U*	*d*, 95% *CI*^d^
	*M*	*SD*	*M*	*SD*	*t*(*df*)	Lower bound	Upper bound		
RSPC	5.05	2.61	6.36	3.02	−2.83^**^ (276)	−2.3	−0.25	3382.50^**^	−0.51 [-0.88, −0.12]
Age	33.59^b^	13.14	39.51	10.84	−2.67^**^ (271)	−9.63	−2.28	3030.50^***^	−0.46 [-0.77, −0.15]
PNCSE-A	1.41	0.99	2.03	1.44	−2.55^*^ (44.08)	−1.12	−0.10	3688.00^**^	−0.57 [-1.05, −0.16]
PNCSE-P	2.49	1.52	3.36	1.60	−3.29^**^ (276)	−1.40	−0.30	3284.50^**^	−0.57 [-0.93, −0.19]
CTQ Sexual Abuse	1.51	0.94	2.03	1.39	−2.27^*^ (43.81)	−1.05	−0.13	3784.00^*^	−0.52 [-0.97, −0.07]
CTQ Emotional Neglect	2.31	0.94	2.44	1.07	−0.80 (276)	−0.52	0.22	4440.50	−0.14 [-0.51, 0.21]
CTQ Emotional Abuse	1.85^c^	0.85	2.17	1.17	−1.65 (44.79)	−0.74	0.07	4049.50	−0.36 [-0.79, 0.06]
CTQ Physical Neglect	1.43	0.55	1.72	0.78	−2.20^*^ (44.41)	−0.55	−0.05	3776.00^*^	−0.49 [-0.94, −0.09]
CTQ Physical Abuse	1.35	0.53	1.66	0.82	−2.28^*^ (43.38)	−0.62	−0.02	3740.00^*^	−0.53 [-0.98, −0.11]

RSPC = Relative Sexual Preference for Children (i.e., self-reported maximum sexual attraction to prepubescent and early/mid pubescent children - self-reported maximum sexual attraction to mature adults). PNCSE-A = Perceived Non-coercive Childhood Sexual Experiences with Adults. PNCSE-P = Perceived Non-coercive Childhood Sexual Experiences with Peers. CTQ = Childhood Trauma Questionnaire. ^*^p < .05, ^**^p < .01, ^***^p < .001.

^a^= based on 1000 bootstrap samples, bias corrected and accelerated (BCa) intervals.

^b^*n* = 234.

^c^*n* = 238.

^d^bootstrap confidence intervals for Cohen’s *d* calculated with the R package bootSE based on 2000 bootstrap resamples.

### Alternative Classification Procedure

As in Study 1, we conducted additional robustness checks by repeating our analyses with a subsample of participants, for whom self-reported and VT-inferred sexual attractions (to pre/peripubescent children vs. postpubescent adults) were in alignment (*N* = 381). Findings are presented in Supplemental Material B (Table S5 for group comparisons, Table S6 for correlational analyses). Again, despite the lowered sample size, we replicated that pedohebephilic men reported more PNCSE-P and CTQ Sexual Abuse and less CTQ Physical Neglect and CTQ Physical Abuse. No significant differences emerged for the remaining scales. Note that one effect (lower rates of CTQ Physical Neglect among pedohebephilic men) was insignificant in the the rank-based Mann-Whitney-*U*-test and based on the 95% BCa *CI*s, even though Cohen’s *d* was higher than in the sample based on self-reported sexual attraction only. Hence, sensitivity tests based on a more conservative classification strategy largely confirmed our previous results based on self-reports.

## Discussion

## Early Sexual Experiences and Pedophebephilic Attraction

While results were generally in line with our expectation (and prior research in forensic and non-forensic settings, see Alanko et al., 2017; Jespersen et al., 2009) that pedohebephilic men report more CSAE than teleiophilic men, we did not find consistent evidence for our hypothesis that they would also report more PNCSE-A. While pedohebephilic men in Study 1 reported more PNCSE-A than teleiophilic men in Study 1, Study 2 revealed an opposite pattern of results. On the one hand, one may argue that Study 2 should be given more weight because of its larger sample size and more diverse teleiophilic sample. On the other hand, it is interesting to note that while pedohebephilic participants in both studies achieved similar scores on the PNCSE-A, teleiophilic participants in Study 2 reported more PNCSE-A than teleiophilic participants in Study 1. Because of the lack of data on the prevalence of PNCSE-A among community adults, it is difficult to know whether or not the Study 2 teleiophilic sample contained an atypically high number of people who have experienced PNCSE-A. Hence, more research is needed to increase our evidence base regarding PNCSE-A and their link to adult sexual attraction.

Study 2 furthermore detected strong differences between pedohebephilic and teleiophilic men regarding the frequency with which they reported to have engaged in PNCSE-P before age 14. This corresponds with previous research showing that pedohebephilic men recall a higher frequency of sexual engagement with peers during their childhood compared to nonpedophilic men (Santtila et al., 2010). Combined with the prior findings that pedohebephilic men recall an earlier age of first ejaculation and masturbation than teleiophilic men (Breiling et al., 2020; Gerwinn et al., 2018) and our result that CSAE is associated with higher rates of reported sexual peer-type activities, Study 2 suggests that early PNCSE-P could be an important etiological precursor of pedohebephilic attraction. This would arguably also correspond better with conditioning theory than the idea that a child “learns” to become sexually aroused in the presence of child bodies through sexual activities with an adult, as a physically mature adult body then would become the conditioned stimulus (see also Santtila et al., 2010). More research based on stronger (e.g., longitudinal) designs is needed to help differentiate whether these early sexual peer activities present a) behavioral expressions of accelerated sexual development that is lived out among peers, b) genuine developmental precursors of pedohebephilia, or c) early signs of already formed sexual interest in prepubescent children among pubescent or post-pubescent sexually precocious boys.

## Non-Sexual Adverse Childhood Experiences and Pedohebephilic Attraction

Regarding other types of maltreatment within the family, the two studies produced mixed results. While Study 1 and 2 found differences between teleiophilic and pedohebephilic men regarding instances of different types of sexual and nonsexual maltreatment, these were usually small or, as in the case of physical neglect or abuse in Study 2, even in the opposite direction from what was expected (and again small). Hence, in the present studies, pedohebephilic men’s early adverse family experiences did not differ markedly from those of teleiophilic men (but note that results based on the CTQ Physical Neglect scale in Study 1 need to be interpreted cautiously due to its insufficient reliability). In the present studies, the effect sizes for the different types of nonsexual childhood adverse experiences were all considerably smaller than those in Gerwinn et al. (2018, emotional abuse: *d* = 0.62, physical abuse: *d* = 0.49, sexual abuse: *d* = 0.50, emotional neglect: *d* = 0.60), with the exception of physical neglect (*d* = 0.26)^2^. Instead, they were more in line with the smaller effect sizes reported in Alanko et al. (2017, Study 2). Of note, Alanko et al. (2017), like us, have studied men outside of a clinical and forensic setting. Gerwinn et al.'s (2018) and Marx et al.’s (2020) participants, however, were recruited predominantly or exclusively from the clinical secondary prevention project network “Don’t offend” (Beier, et al., 2015b) and, hence, must have suffered from relevant impairment due to their sexual interests. Clinical samples of pedohebephilic men can be expected to experience higher rates of distress and mental health problems compared to community samples (Jahnke et al., 2015). These mental health problems may in turn be associated with (and retraced to) childhood maltreatment. Hence, the practice of comparing clinical samples of pedohebephilic men and nonclinical samples in case-control designs may have inadvertently created or exacerbated associations between factors associated with general mental health problems (e.g., childhood adverse experiences) and sexual maturity interests.

## Differences Between Offending and Nonoffending Pedohebephilic Men

We find in both studies that pedohebephilic participants with prior convictions report higher rates of CSAE than teleiophilic men, which is in line with our hypothesis and previous research in online samples of pedohebephilic men (Alanko et al., 2017 Study 1; Bailey et al., 2016). However, results are inconsistent with respect to PNCSE-A, whereby Study 2 finds higher rates and Study 1 lower rates (albeit only descriptively) among pedohebephilic men with as opposed to without prior convictions for child sexual offending. Both studies confirmed that pedohebephilic men who have been convicted for sexual offenses show higher rates of some type of nonsexual childhood adverse experience compared to pedohebephilic participants without convictions (physical neglect and physical abuse in Study 2, and, albeit nonsignificant, physical abuse and emotional neglect in Study 1). The interpretation of these results is complicated by the presence of confounds, namely age and relative sexual preference for children, which vary between the groups (as well as the rate of convictions for sexual offenses which is roughly twice as high in Study 1 as in Study 2).

## Limitations

In the current survey, we asked adults to report experiences dating back many years. Hence, it is to be expected that participants' memories will be biased, vague, or incomplete. People from both the pedohebephilic and the teleiophilic group may also have underreported experiences of adverse childhood experiences. For example, Williams (1994) found that when surveyed as adults, many women cannot recall having experienced the sexual abuse that was, at that time, documented in medical files (but see Korkman et al., 2019 for a reversed potential for biases when relying on third-party reports). Future studies may consider assessing more “objective” information about CSAE alongside subjective measures, such as whether police reports have been filed or whether medical records have documented genital trauma (as in Danese & Widom, 2020). We furthermore recommend that large-scale prospective studies on the effects of childhood trauma (e.g., Fergusson et al., 2013) should inquire about adult sexual attractions of former child victims. Yet, note that PNCSE-A may be less likely to involve acts that physically harm the child (such as penetrative intercourse) and/or that could be detected by medical staff. Moreover, many children do not report CSAE to the authorities (Lahtinen et al., 2018).

Participants' present sexual attraction patterns may also influence their recollection of past events. As people with pedohebephilia are probably aware of lay theories linking childhood adverse experiences to the development of sexual attraction to children (Furnham & Haraldsen, 1998), they may overestimate the frequency of such experiences. Furthermore, not wanting to cause harm while also desiring or engaging in sexual activities with a child may elicit an intense internal conflict, if the adult has experienced similar activities as harmful or unpleasant in his own childhood. This cognitive dissonance could in turn have led some participants to reframe their own CSAE as PNCSE-A. Albeit highly speculative, the mixed results we find for the link between PNCSE-A and pedohebephilia might be due to the sample in Study 2 including a lower rate of men who have been convicted for sexual offenses compared to Study 1 (14% vs. 30%). Yet, note that in theory, cognitive dissonance could also work in opposite ways, and motivate people to re-frame PNCSE-A as CSAE in line with the more predominant idea that child sexual abuse is always experienced as negative or coercive.

Furthermore, although we were mindful of potential confounds in our analyses, causality cannot be inferred from retrospective cross-sectional data. For instance, as sexual activities are more frequent among older (postpubescent) minors, it cannot be ruled out that some of the adverse experiences, as assessed by the CTQ, occurred after sexual attraction to adults or children had already been formed. The same limitation does, of course, also apply to the other subtypes of maltreatment that the CTQ assesses. If adolescents do not develop a sexual attraction to age-appropriate partners, they may become more sensitive to rejection or feel a greater emotional distance from their family. In that case, reported emotional neglect or abuse would be a consequence of emerging pedohebephilic interests, not a causal factor.

Lastly, readers need to keep in mind that we did not assess a representative sample of community men and that our results are therefore tainted by biases associated with convenience sampling. On the one hand, members of the forums or platforms that we included are likely to differ from the general male population on variables of interest. This includes educational achievement, as online samples, including the MTurk workforce, tend to be more educated than the norm population (Paolacci et al., 2010). This has consequences for the generalizability and validity of the present findings, as people with a higher education may be less likely to have been exposed to negative economic or social conditions associated with neglect, abuse, and early sexual behavior (Drake & Pandey, 1996). Particularly users of science or psychology-related forums (as the teleiophilic group in Study 1) can be expected to have higher socioeconomic status and education than people from the general population. This could provide an alternative explanation why teleiophilic participants in Study 1 reported lower rates of abuse or neglect than pedohebephilic participants.

On a related note, people with specific characteristics may also be more motivated to take part in the research than others, which further limits the generalizability of the findings. For instance, it is possible that people who have experienced PNCSE-A or CSAE were more likely to participate because they could relate more to the topics that were studied. Because of these different sources of bias in self-selected samples, it is difficult to reason whether the present studies over- or underestimate the rates of recalled PNCSE-A, PNCSE-P, or adverse childhood experiences in the general population or subgroups of interest.

Given the limited number of forums/support groups for pedohebephilic men, it is also possible that pedohebephilic men with high German and English language proficiency have participated in both B4U-ACT and German language forums and have participated in Study 1 and 2. To handle potential overlap, future research on online samples of pedohebephilic men may include items to detect participants from previous studies on similar topics.

## Implications for Clinical and Research Practice

Between a fifth and a third of the pedohebephilic participants who self-reported PNCSE-A disagreed with every item of the CTQ Sexual Abuse subscale. This noteworthy clinical observation indicates that trauma-focused questionnaires might not be sufficient to detect instances of CSAE in community samples of pedohebephilic men (and also some teleiophilic men, particularly in Study 2). We suppose that this is the case because not all of our male participants feel that the statements about CSAE with adults that imply coercion, exploitation, or unpleasantness do indeed apply to their case. This could result in underreporting of CSAE (that the child may or may not have experienced as coerced and that may or may not have been unpleasant). Memories of PNCSE-A may also explain why pedohebephilic men often express permissive attitudes towards adult-child sex, as long as they assume that the child has not been coerced (Jahnke et al., 2018; Spriggs et al., 2018, but note that Study 2 did not detect significant differences between pedohebephilic and teleiophilic men regarding the frequency of PNCSE-A).

Only a minority of studies besides ours (Felson et al., 2019; Rind & Welter, 2014, 2016) included measures assessing positive memories of CSAE among adolescents or adults from the general population. All of these studies revealed that perceptions of positive or non-coerced CSAE were common (relative to CSAE recalled as having been negative or coerced), at least among boys. Therefore, people conducting clinical interviews in forensic or non-forensic settings should take care to assess these events in a way that allows participants to indicate *any* experiences they might have had. Interview questions need to be framed in a way to not only elicit reports of explicitly negative experiences, but also those which are remembered as positive or as neutral, ambivalent, or indifferent. While legally correct and justifiable based on children’s lack of ability to give informed consent, using terminology such as “sexual abuse” might be misleading for research participants or clients who do not judge these acts to have been abusive, leading them to therefore under-report CSAE.

## Implications for Theory

From a theoretical perspective, the idea that CSAE in itself causes pedohebephilia is recognized as being too simplistic (Garland & Dougher, 1990). The results of the present studies should not let us forget that a large number of pedohebephilic men in both studies did *not* report any CSAE, irrespective of whether these were framed as coercive and unpleasant or not. Moreover, the fact that a non-negligible rate of teleiophilic men (particularly in Study 2) also report CSAE casts further doubt on the simplistic idea that CSAE cause sexual attraction to children. There are likely multiple (i.e., genetic, parental, neurobiological, see Tenbergen et al., 2015) factors that lead to all possible configurations of developing pedohebephilic or teleiophilic sexual attraction and committing sexual offenses against children (or not; Papalia et al., 2018). Even though it seems fair to suppose a multifactorial biopsychosocial etiology model, drawing any conclusions in terms of the relative importance of potential biological and environmental factors to the development of teleiophilic versus pedohebephilic attraction seems premature, based on the present state of knowledge. Nevertheless, even though present effect sizes tend to be small, they are similar to effect sizes reported for biological or cognitive differences between pedohebephilic and teleiophilic men (e.g., *d* = 0.20 for differences in height, *d* = 0.25 for differences in handedness, and *d* = 0.32 for IQ, Cantor et al., 2004; McPhail & Cantor, 2015).

## Outlook

Studying the prevalence, time-lines, characteristics, and correlates of childhood sexual experiences with adults and peers appears to be the next logical step for future research on the developmental origins of sexual attraction to children (as well as the abused-abuser hypothesis; Papalia et al., 2018). Our current studies leave ample room for future research to tackle follow-up questions that we can only speculate about at this preliminary stage: Do pedohebephilic men start masturbating to (selective) memories of CSAE or sexual experiences with others? Did peer-type sexual activities involve others that were younger and/or less physically developed based on preference or on opportunity (indicating attempts to compensate for lacking access to elder partners of choice)? What role does sibling-incest play that is not always experienced as abusive (Tener et al., 2020)? Are there differential effects depending on the sex of the adult involved in PNCSE-A? How do PNCSE-A interact with adult attitudes towards adult-child sex? Does emotional neglect or abuse in the family make children more susceptible to perceive “sexual interaction with an adult an exceptionally positive experience, inasmuch as the child or adolescent may experience needed affection in such a relationship” (Garland & Dougher, 1990, p. 501)? Or do early sexual or non-sexual adverse experiences lead to an earlier onset of sexual activities with (physically less mature) peers because of accelerated pubertal development (Belsky, 2019; Brown et al., 2004)? Only a more fine-grained analysis that is open to different adult interpretations of childhood sexual acts with peers *and* adults will be conducive to elucidate our understanding of the boundary conditions that may contribute to developing pedohebephilic interest.

## Supplemental Material

Supplemental Material - Pedohebephilia and Perceived Non-coercive Childhood Sexual Experiences: Two Non-matched Case-Control Studies Click here for additional data file. Supplemental Material for Pedohebephilia and Perceived Non-coercive Childhood Sexual Experiences: Two Non-matched Case-Control Studies by Sara Jahnke, Alexander F. Schmidt, and Jürgen Hoyer in Sexual Abuse

## Viewing time measure of sexual interest in Study 1

For Study 1, we presented a randomized set of five male and five female pictures of prepubescent children (Tanner stage 1; Tanner, 1990) and postpubescent adults each (Tanner stage 5) from the Not Real People Set (i.e., computer-generated pictures of Caucasian individuals in bathing suits provided by the Pacific Psychological Assessment Cooperation, 2004). Participants rated their attraction to each depicted person in a forced choice format. They were furthermore instructed to respond by clicking on either: “Yes, this is a potential sexual partner for me” versus “No, this is not a potential sexual partner for me” within one second (i.e., speeded VT task, Imhoff et al., 2010, Study 3, but note that we did not exclude trials with longer reaction times). We unobtrusively recorded participants’ reaction times based on the build-in option in SosciSurvey.^3^

We could not record response latencies for 42 participants, which is most likely due to anonymity software preventing the recording of reaction times. We then screened response latency outliers for each single trial with the *adjbox* function from the R package robustbase (Maechler et al., 2019). All outliers were set to missing values (6% of the trials).

For each combination of stimulus Tanner stage (T1 and T5) and sex (male and female), we calculated the average response latency. We then subtracted the maximum average response latency to either male or female Tanner five stimuli from the maximum average response latency to either male or female stimuli Tanner 1 stimuli. Positive differences indicate pedohebephilia, and all participants with difference scores >0 were categorized as pedohebephilic (although technically reflecting pedophilic preferences for prepubescent children). All participants with difference scores <0 were categorized as teleiophilic. Following these steps, we were able to classify 67 men as pedohebephilic and 90 men as teleiophilic.

## Viewing time measure of sexual interest in Study 2

In Study 2, we used pictures of people from all Tanner stages (i.e., Tanner stages 1–5) from the Not Real People set (Pacific Psychological Assessment Cooperation, 2004). To shorten test duration based on the larger stimulus set compared to Study 1, we showed four (instead of 5) pictures for each combination of stimulus sex (male/female) and Tanner stage (T1, T2, T3, T4, T5).

For 110 participants, it was not possible to record response latency data. Following procedures in Study 1, we deleted 4% of recorded trials because the *adjbox* function marked them as outliers. We calculated difference scores between maximum response latencies to either male or female stimuli for each of the five Tanner stages. Hence, we subtracted maximum average response latencies to pictures Tanner stages 4 and 5 (late stages of puberty and post-puberty) from Tanner stages 1, 2 and 3 (pre-puberty and early to medium stages of puberty). Again, positive scores indicated pedohebephilia, and all participants with difference scores >0 were categorized as pedohebephilic. Mean difference scores <0 were classified as teleiophilic. This led to the categorization of 60 participants as pedophilic (who showed higher response latencies for stimuli in Tanner stage 1 than in Tanner stages 2–5) and 119 as hebephilic (who showed higher response latencies for stimuli in Tanner stages 2 and 3 than for Tanner stages 1, 4, and 5). A total of 326 men was classified as teleiophilic (who showed higher values for stimuli in Tanner stages 4 and 5 than stimuli in Tanner stages 1–3).

## Notes

1 Note that there is a distinction between the individual *perception of consent* and *informed consent*. Informed consent to sexual acts would imply that a child does not only agree to engage in sex but also has a deeper understanding of sexual acts and relationships, as well as the freedom to say no (Finkelhor, 1979). In the present paper, when we assess or discuss the retrospective perception of childhood consent when engaging in sex with adults, we are not implying that children can give true or meaningful informed consent to sexual acts with adults.

2 Formula: $\mathrm{d=}M_{1}+M_{2}/SD_{\mathrm{pooled}}$, *M*s and *SD*s for the comparison between nonoffending pedohebephilic men (*n* = 76) and control participants without pedohebephilia and no history of sexual offending (*n* = 144) retrieved from Gerwinn et al. (2018).

3 The reported effects and convergent validity results speak to the feasibility of these proof-of-concept studies with VT measures used in freely available survey software (i.e., SosciSurvey).

## References

[bibr1-10790632221098341] Alanko K. Gunst A. Mokros A. Santtila P. (2016). Genetic variants associated with male pedophilic sexual interest. The Journal of Sexual Medicine, 13(5), 835–842. 10.1016/j.jsxm.2016.02.170 27114195

[bibr2-10790632221098341] Alanko K. Schmidt A. F. Neutze J. Bergen E. Santtila P. Osterheider M. (2017). Male sexual interest in and offending against children: The abused-abuser hypothesis. Journal of Forensic Psychology Research and Practice, 17(2), 128–144. 10.1080/24732850.2017.1286544

[bibr3-10790632221098341] Araji S. Finkelhor D. (1985). Explanations of pedophilia: Review of empirical research. Bulletin of the American Academy of Psychiatry and the Law, 13(1), 17–37. 3888317

[bibr4-10790632221098341] Babchishin K. M. Seto M. C. Fazel S. Långström N. (2019). Are there early risk markers for pedophilia? A nationwide case-control study of child sexual exploitation material offenders. Journal of Sex Research, 56(2), 203–212. 10.1080/00224499.2018.1492694 30064261 PMC6225987

[bibr5-10790632221098341] Bagley C. Wood M. Young L. (1994). Victim to abuser: Mental health and behavioral sequels of child sexual abuse in a community survey of young adult males. Child Abuse & Neglect, 18(8), 683–697. 10.1016/0145-2134(94)90018-3 7953908

[bibr6-10790632221098341] Bailey J. M. Bernhard P. A. Hsu K. J. (2016). An Internet study of men sexually attracted to children: Correlates of sexual offending against children. Journal of Abnormal Psychology, 125(7), 989–1000. 10.1037/abn0000213 27732028

[bibr7-10790632221098341] Bártová K. Androvičová R. Krejčová L. Weiss P. Klapilová K. (2021). The prevalence of paraphilic interests in the Czech population: Preference, arousal, the use of pornography, fantasy, and behavior. The Journal of Sex Research, 58(1), 86–96. 10.1080/00224499.2019.1707468 31916860

[bibr8-10790632221098341] Beier K. M. Amelung T. Kuhle L. Grundmann D. Scherner G. Neutze J. (2015a). Hebephilia as a sexual disorder. Fortschritte Der Neurologie · Psychiatrie, 83(02), e1–e9. 10.1055/s-0034-1398960 25723776

[bibr9-10790632221098341] Beier K. M. Grundmann D. Kuhle L. F. Scherner G. Konrad A. Amelung T. (2015b). The German Dunkelfeld Project: A pilot study to prevent child sexual abuse and the use of child abusive images. The Journal of Sexual Medicine, 12(2), 529–542. 10.1111/jsm.12785 25471337

[bibr10-10790632221098341] Belsky J. (2019). Early-life adversity accelerates child and adolescent development. Current Directions in Psychological Science, 28(3), 241–246. 10.1177/0963721419837670

[bibr11-10790632221098341] Bernstein D. P. Stein J. A. Newcomb M. D. Walker E. Pogge D. Ahluvalia T. Stokes J. Handelsman L. Medrano M. Desmond D. Zule W. (2003). Development and validation of a brief screening version of the Childhood Trauma Questionnaire. Child Abuse & Neglect, 27(2), 169–190. 10.1016/S0145-2134(02)00541-0 12615092

[bibr12-10790632221098341] Breiling L. Rettenberger M. Turner D. (2020). The relevance of sexual biographies in individuals convicted of child sexual abuse offenses for the development of pedosexual interests and sexual recidivism. Sexual Offending: Theory, Research, and Prevention, 15(1), 1–26. 10.5964/sotrap.3711

[bibr13-10790632221098341] Brown J. Cohen P. Chen H. Smailes E. Johnson J. G. (2004). Sexual trajectories of abused and neglected youths. Journal of Developmental & Behavioral Pediatrics, 25(2), 77–82. 10.1097/00004703-200404000-00001 15083128

[bibr14-10790632221098341] Buhrmester M. Kwang T. Gosling S. D. (2011). Amazon’s Mechanical Turk: A new source of inexpensive, yet high-quality, data? Perspectives on Psychological Science, 6(1), 3–5. 10.1177/1745691610393980 26162106

[bibr15-10790632221098341] Burton D. L. Meezan W. (2004). Revisiting recent research on social learning theory as an etiological proposition for sexually abusive male adolescents. Journal of Evidence-Based Social Work, 1(1), 41–80. 10.1300/J394v01n01_04 28879816

[bibr16-10790632221098341] Cantor J. M. Blanchard R. Christensen B. K. Dickey R. Klassen P. E. Beckstead A. L. Blak T. Kuban M. E. (2004). Intelligence, memory, and handedness in pedophilia. Neuropsychology, 18(1), 3–14. 10.1037/0894-4105.18.1.3 14744183

[bibr17-10790632221098341] Cohen J (1992). A power primer. Psychological Bulletin, 112(1), 155–159. 10.1037//0033-2909.112.1.155 19565683

[bibr18-10790632221098341] Cohen L. J. Forman H. Steinfeld M. Fradkin Y. Frenda S. Galynker I. (2010). Comparison of childhood sexual histories in subjects with pedophilia or opiate addiction and healthy controls: Is childhood sexual abuse a risk factor for addictions? Journal of Psychiatric Practice®, 16(6), 394–404. 10.1097/01.pra.0000390758.27451.79 21107144

[bibr19-10790632221098341] Craissati J. McClurg G. Browne K. (2002). Characteristics of perpetrators of child sexual abuse who have been sexually victimized as children. Sexual Abuse: A Journal of Research and Treatment, 14(3), 225–239. 10.1023/A:1015318408395 12087684

[bibr20-10790632221098341] Danese A. Widom C. S. (2020). Objective and subjective experiences of child maltreatment and their relationships with psychopathology. Nature Human Behaviour, 4(8), 811–818. 10.1038/s41562-020-0880-3 32424258

[bibr21-10790632221098341] Dombert B. Schmidt A. F. Banse R. Briken P. Hoyer J. Neutze J. Osterheider M. (2016). How common is men’s self-reported sexual interest in prepubescent children? The Journal of Sex Research, 53(2), 214–223. 10.1080/00224499.2015.1020108 26241201

[bibr22-10790632221098341] Drake B. Pandey S. (1996). Understanding the relationship between neighborhood poverty and specific types of child maltreatment. Child Abuse & Neglect, 20(11), 1003–1018. 10.1016/0145-2134(96)00091-9 8958452

[bibr23-10790632221098341] Faul F. Erdfelder E. Lang A.-G. Buchner A. (2007). G*Power 3: A flexible statistical power analysis program for the social, behavioral, and biomedical sciences. Behavior Research Methods, 39(2), 175–191. 10.3758/bf03193146 17695343

[bibr24-10790632221098341] Fazio RL (2018). Toward a neurodevelopmental understanding of pedophilia. The Journal of Sexual Medicine, 15(9), 1205–1207. 10.1016/j.jsxm.2018.04.631 29861362

[bibr25-10790632221098341] Feelgood S. Hoyer J. (2008). Child molester or paedophile? Sociolegal versus psychopathological classification of sexual offenders against children. Journal of Sexual Aggression, 14(1), 33–43. 10.1080/13552600802133860

[bibr26-10790632221098341] Felson R. B. Savolainen J. Fry S. Whichard C. Ellonen N. (2019). Reactions of boys and girls to sexual abuse and to sexual encounters with peers. Journal of Youth and Adolescence, 48(10), 1869–1882. 10.1007/s10964-019-01111-1 31478119

[bibr27-10790632221098341] Fergusson D. M. McLeod G. F. H. Horwood L. J. (2013). Childhood sexual abuse and adult developmental outcomes: Findings from a 30-year longitudinal study in New Zealand. Child Abuse & Neglect, 37(9), 664–674. 10.1016/j.chiabu.2013.03.013 23623446

[bibr28-10790632221098341] Finkelhor D. (1979). What’s wrong with sex between adults and children? Ethics and the problem of sexual abuse. American Journal of Orthopsychiatry, 49(4), 692–697. 10.1111/j.1939-0025.1979.tb02654.x 495708

[bibr29-10790632221098341] Finkelhor D. Ormrod R. K. Turner H. A. (2007). Poly-victimization: A neglected component in child victimization. Child Abuse & Neglect, 31(1), 7–26. 10.1016/j.chiabu.2006.06.008 17224181

[bibr30-10790632221098341] Finkelhor D. Shattuck A. Turner H. A. Hamby S. L. (2014). The lifetime prevalence of child sexual abuse and sexual assault assessed in late adolescence. Journal of Adolescent Health, 55(3), 329–333. 10.1016/j.jadohealth.2013.12.026 24582321

[bibr31-10790632221098341] Freund K. Kuban M. (1994). The basis of the abused abuser theory of pedophilia: A further elaboration on an earlier study. Archives of Sexual Behavior, 23(5), 553–563. 10.1007/BF01541497 7998815

[bibr32-10790632221098341] Furnham A. Haraldsen E. (1998). Lay theories of etiology and “cure” for four types of paraphilia: Fetishism; pedophilia; sexual sadism; and voyeurism. Journal of Clinical Psychology, 54(5), 689–700. 10.1002/(sici)1097-4679(199808)54:5<689::aid-jclp15>3.0.co;2-9 9696119

[bibr33-10790632221098341] Garland R. J. Dougher M. J. (1990). The abused/abuser hypothesis of child sexual abuse: A critical review of theory and research. In Pedophilia (pp. 488–509). Springer. 10.1007/978-1-4613-9682-6_20

[bibr34-10790632221098341] Gerwinn H. Weiß S. Tenbergen G. Amelung T. Födisch C. Pohl A. Massau C. Kneer J. Mohnke S. Kärgel C. Wittfoth M. Jung S. Drumkova K. Schiltz K. Walter M. Beier K. M. Walter H. Ponseti J. Schiffer B. Kruger T. H. C. (2018). Clinical characteristics associated with paedophilia and child sex offending–Differentiating sexual preference from offence status. European Psychiatry, 51, 74–85. 10.1016/j.eurpsy.2018.02.002 29625377

[bibr35-10790632221098341] Grady M. D. Levenson J. S. (2021). Prevalence rates of adverse childhood experiences in a sample of minor-attracted persons: A comparison study. Traumatology, 27(2), 227–235. 10.1037/trm0000273

[bibr36-10790632221098341] Hershkowitz I. (2014). Sexually intrusive behavior among alleged CSA male victims: A prospective study. Sexual Abuse, 26(3), 291–305. 10.1177/1079063213486937 23698743

[bibr37-10790632221098341] Houtepen J. A. Sijtsema J. J. Bogaerts S. (2015). Being sexually attracted to minors: Sexual development, coping with forbidden feelings, and relieving sexual arousal in self-identified pedophiles. Journal of Sex and Marital Therapy, 42(1), 48–69. 10.1080/0092623X.2015.1061077 26098192

[bibr38-10790632221098341] Imhoff R. Schmidt A. F. Nordsiek U. Luzar C. Young A. W. Banse R. (2010). Viewing Time effects revisited: Prolonged response latencies for sexually attractive targets under restricted task conditions. Archives of Sexual Behavior, 39(6), 1275–1288. 10.1007/s10508-009-9595-2 20198414

[bibr39-10790632221098341] Jahnke S. Malón A. (2019). How pedohebephilic men think about adult-child sex: Effects of child gender and physical maturity. Psychology, Crime & Law, 25(1), 90–107. 10.1080/1068316X.2018.1503665

[bibr40-10790632221098341] Jahnke S. Schmidt A. F. Geradt M. Hoyer J. (2015). Stigma-related stress and its correlates among men with pedophilic sexual interests. Archives of Sexual Behavior, 44(8), 2173–2187. 10.1007/s10508-015-0503-7 25933669

[bibr42-10790632221098341] Jahnke S. Schmidt A. F. Klöckner A. Hoyer J. (2022). Neurodevelopmental differences, pedohebephilia, and sexual offending: Findings from two online surveys. Archives of Sexual Behavior, 51(2), 849–866. 10.1007/s10508-021-02228-w 34993718 PMC8888371

[bibr43-10790632221098341] Jahnke S. Schmitt S. Malón A. (2018). What if the child appears to enjoy it? Moral attitudes toward adult–child sex among men with and without pedohebephilia. The Journal of Sex Research, 55(7), 927–938. 10.1080/00224499.2016.1271101 28139141

[bibr44-10790632221098341] Jespersen A. F. Lalumière M. L. Seto M. C. (2009). Sexual abuse history among adult sex offenders and non-sex offenders: A meta-analysis. Child Abuse & Neglect, 33(3), 179–192. 10.1016/j.chiabu.2008.07.004 19327831

[bibr45-10790632221098341] Kirby K. N. Gerlanc D. (2013). BootES: An R package for bootstrap confidence intervals on effect sizes. Behavior Research Methods, 45(4), 905–927. 10.3758/s13428-013-0330-5 23519455

[bibr46-10790632221098341] Klucken T. Schweckendiek J. Merz C. J. Tabbert K. Walter B. Kagerer S. Vaitl D. Stark R. (2009). Neural activations of the acquisition of conditioned sexual arousal: Effects of contingency awareness and sex. The Journal of Sexual Medicine, 6(11), 3071–3085. 10.1111/j.1743-6109.2009.01405.x 19656273

[bibr47-10790632221098341] Korkman J. Antfolk J. Fagerlund M. Santtila P. (2019). The prevalence of unfounded suspicions of child sexual abuse in Finland. Nordic Psychology, 71(1), 39–50. 10.1080/19012276.2018.1470554

[bibr48-10790632221098341] Lahtinen H.-M. Laitila A. Korkman J. Ellonen N. (2018). Children’s disclosures of sexual abuse in a population-based sample. Child Abuse & Neglect, 76, 84–94. 10.1016/j.chiabu.2017.10.011 29096161

[bibr49-10790632221098341] Leiner D. J. (2014). SoSci survey (version 2.5.00—I). https://www. soscisurvey.de.

[bibr50-10790632221098341] Levenson J. S. Willis G. M. Prescott D. S. (2016). Adverse childhood experiences in the lives of male sex offenders: Implications for tauma-informed care. Sexual Abuse, 28(4), 340–359. 10.1177/1079063214535819 24872347

[bibr51-10790632221098341] MacDonald K. Thomas M. L. MacDonald T. M. Sciolla A. F. (2015). A perfect childhood? Clinical correlates of minimization and denial on the Childhood Trauma Questionnaire. Journal of Interpersonal Violence, 30(6), 988–1009. 10.1177/0886260514539761 24981003

[bibr52-10790632221098341] Maechler M. Rousseeuw P. Croux C. Todorov V. Ruckstuhl A. Salibian-Barrera M. Verbeke T. Koller M. Conceicao E. L. T. di Palma M. A. (2019). robustbase: Basic robust statistics (0.93-5) [Computer software]. https://CRAN.R-project.org/package=robustbase

[bibr53-10790632221098341] Malón A. (2017). Adult-child sex and the demands of virtuous sexual morality. Sexuality & Culture, 21(1), 247–269. 10.1007/s12119-016-9392-8

[bibr54-10790632221098341] Marx C. M. Tibubos A. N. Brähler E. Beutel M. E. (2020). Experienced childhood maltreatment in a sample of pedophiles: Comparisons with patients of a psychosomatic outpatient clinic and the general population. The Journal of Sexual Medicine, 17(5), 985–993. 10.1016/j.jsxm.2020.01.019 32089484

[bibr55-10790632221098341] McPhail I. V. Cantor J. M. (2015). Pedophilia, height, and the magnitude of the association: A research note. Deviant Behavior, 36(4), 288–292. 10.1080/01639625.2014.935644

[bibr56-10790632221098341] Nunes K. L. Hermann C. A. Renee Malcom J. Lavoie K. (2013). Childhood sexual victimization, pedophilic interest, and sexual recidivism. Child Abuse & Neglect, 37(9), 703–711. 10.1016/j.chiabu.2013.01.008 23517571

[bibr57-10790632221098341] Okami P. (1991). Self-reports of “positive” childhood and adolescent sexual contacts with older persons: An exploratory study. Archives of Sexual Behavior, 20(5), 437–457. 10.1007/BF01542407 1747040

[bibr58-10790632221098341] Pacific Psychological Assessment Cooperation (2004). The Not-Real People stimulus set for assessment of sexual interest.

[bibr59-10790632221098341] Paolacci G. Chandler J. Ipeirotis P. G. (2010). Running experiments on amazon mechanical turk. Judgment and Decision Making, 5(5), 411–419.

[bibr60-10790632221098341] Papalia N. Luebbers S. Ogloff J. R. P. (2018). Child sexual abuse and the propensity to engage in criminal behaviour: A critical review and examination of moderating factors. Aggression and Violent Behavior, 43, 71–89. 10.1016/j.avb.2018.10.007

[bibr61-10790632221098341] Pedneault C. I. Hilgard J. Pettersen C. Hermann C. A. White K. Nunes K. L. (2021). How well do indirect measures assess sexual interest in children? A meta-analysis. Journal of Consulting and Clinical Psychology, 89(4), 350–363. 10.1037/ccp0000627 33734722

[bibr62-10790632221098341] Pfaus J. G. Kippin T. E. Centeno S. (2001). Conditioning and sexual behavior: A review. Hormones and Behavior, 40(2), 291–321. 10.1006/hbeh.2001.1686 11534994

[bibr63-10790632221098341] Ray J. J. (1984). The reliability of short social desirability scales. Journal of Social Psychology, 123(1), 133–134. 10.1080/00224545.1984.9924522

[bibr64-10790632221098341] Rind B. (2021). First sexual intercourse in the Irish study of sexual health and relationships: Current functioning in relation to age at time of experience and partner age. Archives of Sexual Behavior, 50(1), 289–310. 10.1007/s10508-020-01721-y 32430871

[bibr65-10790632221098341] Rind B. (2022). Reactions to minor-older and minor-peer sex as a function of personal and situational variables in a Finnish nationally representative student sample. Archives of Sexual Behavior, 51(2), 961–985. 10.1007/s10508-021-02224-0 35018515

[bibr66-10790632221098341] Rind B. Tromovitch P. Bauserman R. (1998). A meta-analytic examination of assumed properties of child sexual abuse using college samples. Psychological Bulletin, 124(1), 22–53. 10.1037/0033-2909.124.1.22 9670820

[bibr67-10790632221098341] Rind B. Welter M. (2014). Enjoyment and emotionally negative reactions in minor–adult versus minor–peer and adult–adult first postpubescent coitus: A secondary analysis of the Kinsey data. Archives of Sexual Behavior, 43(2), 285–297. 10.1007/s10508-013-0186-x 24233327

[bibr68-10790632221098341] Rind B. Welter M. (2016). Reactions to first postpubertal coitus and first male postpubertal same-sex experience in the Kinsey sample: Examining assumptions in German law concerning sexual self-determination and age cutoffs. International Journal of Sexual Health, 28(2), 117–128. 10.1080/19317611.2016.1150379

[bibr69-10790632221098341] Santtila P. Antfolk J. Räfså A. Hartwig M. Sariola H. Sandnabba N. K. Mokros A. (2015). Men’s sexual interest in children: One-year incidence and correlates in a population-based sample of Finnish male twins. Journal of Child Sexual Abuse, 24(2), 115–134. 10.1080/10538712.2015.997410 25747416

[bibr70-10790632221098341] Santtila P. Mokros A. Hartwig M. Varjonen M. Jern P. Witting K. von der Pahlen B. Sandnabba N. K. (2010). Childhood sexual interactions with other children are associated with lower preferred age of sexual partners including sexual interest in children in adulthood. Psychiatry Research, 175(1-2), 154–159. 10.1016/j.psychres.2008.10.021 19969376

[bibr71-10790632221098341] Schmidt A. F. Babchishin K. M. Lehmann R. J. B. (2017). A meta-analysis of viewing time measures of sexual interest in children. Archives of Sexual Behavior, 46(1), 287–300. 10.1007/s10508-016-0806-3 27543106

[bibr72-10790632221098341] Seto M. C. (2017). The puzzle of male chronophilias. Archives of Sexual Behavior, 46(1), 3–22. 10.1007/s10508-016-0799-y 27549306

[bibr73-10790632221098341] Seto M. C. (2018). Pedophilia and sexual offending against children: Theory, assessment, and intervention (2nd edition). American Psychological Association. https://www.apa.org/pubs/books/4317491

[bibr74-10790632221098341] Singy P (2015). Hebephilia: A postmortem dissection. Archives of Sexual Behavior, 44(5), 1109–1116. 10.1007/s10508-015-0542-0 25894647

[bibr75-10790632221098341] Spriggs S. A. Cohen L. J. Valencia A. Yaseen Z. S. Galynker I. I. (2018). Qualitative analysis of attitudes toward adult-child sexual activity among minor-attracted persons. Journal of Sex & Marital Therapy, 44(0), 787–799. 10.1080/0092623X.2018.1474406 29741472

[bibr76-10790632221098341] Statistisches Bundesamt (2019, February 18). *20- bis 24-Jährige: Mehr als die Hälfte hat Abitur*. Statistisches Bundesamt. https://www.destatis.de/DE/Presse/Pressemitteilungen/2019/02/PD19_055_213.html

[bibr77-10790632221098341] Stephens S. Seto M. C. Goodwill A. M. Cantor J. M. (2017). Evidence of construct validity in the assessment of hebephilia. Archives of Sexual Behavior, 46(1), 301–309. 10.1007/s10508-016-0907-z 27900492

[bibr78-10790632221098341] Streiner DL (2015). Best (but oft-forgotten) practices: The multiple problems of multiplicity—whether and how to correct for many statistical tests. The American Journal of Clinical Nutrition, 102(4), 721–728. 10.3945/ajcn.115.113548 26245806

[bibr79-10790632221098341] Tanner J. M. (1990). Foetus into man: Physical growth from conception to maturity. Harvard University Press.

[bibr80-10790632221098341] Tenbergen G. Wittfoth M. Frieling H. Ponseti J. Walter M. Walter H. Beier K. M. Schiffer B. Kruger T. H. C. (2015). The neurobiology and psychology of pedophilia: Recent advances and challenges. Frontiers in Human Neuroscience, 9, 344. 10.3389/fnhum.2015.00344 26157372 PMC4478390

[bibr81-10790632221098341] Tener D. Tarshish N. Turgeman S. (2020). Victim, perpetrator, or just my brother?” Sibling sexual abuse in large families: A child advocacy center study. Journal of Interpersonal Violence, 35(21–22), 4887–4912. 10.1177/0886260517718831 29294821

[bibr82-10790632221098341] Trickett P. K. Noll J. G. Putnam F. W. (2011). The impact of sexual abuse on female development: Lessons from a multigenerational, longitudinal research study. Development and Psychopathology, 23(2), 453–476. 10.1017/S0954579411000174 23786689 PMC3693773

[bibr83-10790632221098341] Ward T. Hudson S. M. Marshall W. L. Siegert R. (1995). Attachment style and intimacy deficits in sexual offenders: A theoretical framework. Sexual Abuse, 7(4), 317–335. 10.1177/107906329500700407

[bibr84-10790632221098341] Williams L. M. (1994). Recall of childhood trauma: A prospective study of women’s memories of child sexual abuse. Journal of Consulting and Clinical Psychology, 62(6), 1167–1176. 10.1037/0022-006X.62.6.1167 7860814

[bibr85-10790632221098341] Wingenfeld K. Spitzer C. Mensebach C. Grabe H. Hill A. Gast U. Schlosser N. Höpp H. Beblo T. Driessen M. (2010). Die deutsche Version Des Childhood Trauma Questionnaire (CTQ): Erste Befunde zu den psychometrischen Kennwerten. PPmP Psychotherapie Psychosomatik Medizinische Psychologie, 60(11), 442–450. 10.1055/s-0030-1247564 20200804

[bibr86-10790632221098341] Witt A. Brown R. C. Plener P. L. Brähler E. Fegert J. M. (2017). Child maltreatment in Germany: Prevalence rates in the general population. Child and Adolescent Psychiatry and Mental Health, 11(1), 47. 10.1186/s13034-017-0185-0 28974983 PMC5621113

[bibr87-10790632221098341] Wurtele S. K. Simons D. A. Moreno T. (2014). Sexual interest in children among an online sample of men and women: Prevalence and correlates. Sexual Abuse, 26(6), 546–568. 10.1177/1079063213503688. 24215791

[bibr88-10790632221098341] Wurtele S. K. Simons D. A. Parker L. J. (2018). Understanding men’s self-reported sexual interest in children. Archives of Sexual Behavior, 47(8), 2255–2264. 10.1007/s10508-018-1173-z 29667036

