# Supplementary material for: Pedohebephilia and Perceived Non-coercive Childhood Sexual Experiences: Two Non-matched Case-Control Studies
Source: Sex Abuse. 2022 May 12;35(3):340–74. doi: 10.1177/10790632221098341 (PMC10041574; doi:10.1177/10790632221098341)
Supplement: Supplemental Material - Pedohebephilia and Perceived Non-coercive Childhood Sexual Experiences: Two Non-matched Case-Control Studies [file sj-pdf-1-sax-10.1177_10790632221098341.pdf]

Supplemental Material A: Differences between pedophilic, hebephilic, and teleiophilic participants.

Table S1. Comparisons between the self-reported pedophilia, hebephilia, and teleiophilia group (Study 1)

	Pedophilia (P) (*n* = 53)		Hebephilia (H) (*n* = 48)		Teleiophilia (T) (*n* = 89)		ANOVA test statistics	Significant post hoc comparisons, 95% *CI* of the mean difference
	M	*SD*	*M*	*SD*	*M*	*SD*	*F* (*df*)	
PNCSE-A	1.58	1.26	1.28 [a]	.66	1.08	.38	6.90* (186)	P vs. T: [0.08, 0.93]
CTQ Sexual Abuse	1.31	.57	1.44	.95	1.08	.27	6.25* (187)	P vs. T: [0.03, 0.43]<br>H vs. T: [0.02, 0.70]
CTQ Emotional Neglect	2.36	1.03	2.26	1.02	1.98	.87	3.09* (187)	none [c]
CTQ Emotional Abuse	1.86	.84	1.68	.80	1.54	.67	2.86 (187)	-
CTQ Physical Neglect	1.58	.56	1.55	.64	1.36	.43	3.75* (187)	P vs. T: [0.01, 0.44]
CTQ Physical Abuse	1.25 [b]	.64	1.40	.80	1.12	.34	3.75* (186)	none
Age	36.17	11.21	39.02	13.63	32.48	11.39	4.93* (187)	H vs. T: [1.03, 12.05] [c]

Note. Post hoc tests were only carried out for significant omnibus tests. If not otherwise noted, post hoc tests were corrected with Games-Howell's test for multiple comparisons because of unequal variances. PNCSE-A = Perceived Non-coercive Childhood Sexual Experiences with Adults. CTQ = Childhood Trauma Questionnaire.

[a] $n = 47$

[b] $n = 52$

[c] Tukey-HSD because the error variance of the dependent variable was equal across the three groups.

* $p < .05$

Table S2. Comparisons between the self-reported pedophilia, hebephilia, and teleiophilia group (Study 2)

	Pedophilia (P) (*n* = 137)		Hebephilia (H) (*n* = 141)		Teleiophilia (T) (*n* = 316)		ANOVA test statistics	Significant post hoc comparisons, 95% *CI* of the mean difference
	M	*SD*	*M*	*SD*	*M*	*SD*	*F* (*df*)	
PNCSE-A	1.45	1.06	1.55	1.11	1.42	.99	0.76 (591)	-
PNCSE-P	2.45	1.53	2.76	1.58	1.88	1.26	21.82** (591)	P vs. T: [0.23, 0.93]<br>H vs. T: [0.53, 1.24]
CTQ Sexual Abuse	1.54	1.05	1.62	1.01	1.30	0.71	8.09** (591)	P vs. T: [0.02, 0.48]<br>H vs. T: [0.10, 0.54]
CTQ Emotional Neglect	2.27	0.98	2.38	0.94	2.16	0.93	2.76 (591)	-
CTQ Emotional Abuse	1.83	0.81	1.96[a]	0.99	1.82	0.90	1.26 (590)	-
CTQ Physical Neglect	1.43	0.55	1.51	0.64	1.58	0.66	2.62 (591)	-
CTQ Physical Abuse	1.40	0.59	1.38	0.59	1.51	0.72	2.61 (591)	-
Age	33.72[b]	13.42	35.14[c]	12.55	35.23[d]	11.14	0.80 (587)	-

Note. Post hoc tests were only carried out for significant omnibus tests. All post hoc tests were corrected with Games-Howell's test for multiple comparisons because of unequal variances. PNCSE-A = Perceived Non-coercive Childhood Sexual Experiences with Adults. PNCSE-P = Perceived Non-coercive Childhood Sexual Experiences with Peers. CTQ = Childhood Trauma Questionnaire.

* $p < .05$, ** $p < .01$

[a] $n = 140$

[b] $n = 135$

[c] $n = 138$

[d] $n = 317$

Supplemental Material B: Results for classification based on concordant VT and self-report

Table S3. Comparison of self-reported pedohebephilic and teleiophilic participants (Study 1).

Variables	Pedohebephilia (n = 53)		Teleiophilia (n = 74)			Mean difference 95% CI[b]		Mann-Whitney U	d, 95% CI [c]
	M	SD	M	SD	$t(df)$	Lower bound	Upper bound		
Self-reported attraction to prepubescents	7.87	2.52	1.18	0.53	19.04*** (55.34)	6.02	7.28	18.00***	3.99 [3.13, 5.19]
Self-reported attraction to early – mid pubescents	8.00	2.19	2.30	1.73	16.39*** (125)	4.99	6.48	160.00***	2.95 [2.23, 3.74]
Self-reported attraction to adults	3.43	2.45	9.54	0.98	-17.21*** (64.09)	-6.76	-5.44	69.00***	-3.50 [-4.38, -2.71]
Age	39.68	13.64	31.81	11.67	3.40** (101.06)	3.37	12.69	1201.50***	0.63 [0.23, 1.02]
PNCSE-A	1.40	1.01	1.04	0.26	2.51* (56.94)	0.11	0.67	1712.50**	0.52 [0.23, 0.79]
CTQ Sexual Abuse	1.42	0.95	1.08	0.30	2.46* (59.21)	0.08	0.64	1461.50***	0.51 [0.19, 0.78]
CTQ Emotional Neglect	2.44	1.11	1.90	0.86	2.94** (94.1)	0.16	0.92	1393.50**	0.55 [0.16, 0.91]
CTQ Emotional Abuse	1.78	0.93	1.51	0.59	2.00* (125)	-0.01	0.60	1625.50	0.36 [0.01, 0.69] [c]
CTQ Physical Neglect	1.60	0.63	1.33	0.43	2.72** (85.01)	0.07	0.50	1394.50**	0.52 [0.15, 0.87]
CTQ Physical Abuse	1.35 [a]	0.79	1.13	0.37	1.83 (66.47)	0.01	0.47	1635.50	0.37 [-0.02, 0.68]

Note. Participants with an equally strong attraction to adults and prepubescents or early – mid pubescent were excluded from the analyses. PNCSE-A = Perceived Non-coercive Childhood Sexual Experiences with Adults. CTQ = Childhood Trauma Questionnaire.

*p < .05, **p < .01, ***p < .001

[a] n = 52

[b] based on 1,000 bootstrap samples, bias corrected and accelerated (BCa) intervals

[c] bootstrap confidence intervals for Cohen's d calculated with the R package bootSE based on 2000 bootstrap resamples. As these calculations are based on different bootstrap samples than the BCa intervals of the mean difference, it is possible that statistical significance changes.

Table S4. Intercorrelations of study variables (Study 1, N = 126-127).

Variables	PNCSE_A	CTQ SA	CTQ EN	CTQ EA	CTQ PN	CTQ PA	Age	RSPC
PNCSE_A	-	$.34^{***}$	.12	.09	.09	.07	-	$.26^{**}$
CTQ Sexual Abuse (SA)	$.36^{***}$	-	$.29^{***}$	$.39^{***}$	$.27^{**}$	$.45^{***}$	-	$.22^{*}$
CTQ Emotional Neglect (EN)	$.19^{*}$	$.33^{***}$	-	$.58^{***}$	$.58^{***}$	$.46^{***}$	-	$.19^{*}$
CTQ Emotional Abuse (EA)	.10	$.39^{***}$	$.55^{***}$	-	$.37^{***}$	$.60^{***}$	-	.16
CTQ Physical Neglect (PN)	.14	$.30^{***}$	$.63^{***}$	$.38^{***}$	-	$.45^{***}$	-	.16
CTQ Physical Abuse (PA)	.11	$.47^{***}$	$.51^{***}$	$.60^{***}$	$.49^{***}$	-	-	.08
Age	$.20^{*}$	.16	$.44^{***}$	.08	$.29^{**}$	$.25^{**}$	-	-
Relative Sexual Preference for Children (RSPC)[a]	$.30^{***}$	$.25^{**}$	$.28^{**}$	$.18^{*}$	$.23^{**}$	.14	$.28^{**}$	-

Note. Variables below the diagonal are bivariate correlations, variables above the diagonal are partial correlations (controlling for the effect of age). PNCSE_A = Perceived Non-coercive Childhood Sexual Experiences with Adults. CTQ = Childhood Trauma Questionnaire.

$^{*}p < .05$, $^{**}p < .01$, $^{***}p < .001$

[a] Self-reported maximum sexual attraction to prepubescent and early-to-mid pubescent children - self-reported maximum sexual attraction to mature adults. Note that we used relative strength of pedohebephilic attraction as a continuous indicator to maximize power and sample size (as participants with an equally strong self-reported attraction to children and adults were included and the full variation across the sample could be utilized).

Table S5. Comparison of self-reported pedohebephilic and teleiophilic participants (Study 2).

Variables	Pedohebephilia (n = 114)		Teleiophilia (n = 267)			Mean difference 95% *CI* [c]		Mann-Whitney *U*	*d*, 95% *CI* [d]
	M	*SD*	*M*	*SD*	$t(df)$	Lower bound	Upper bound		
Self-reported attraction to prepubescents	8.46	2.26	1.15	0.75	33.69*** (123.67)	6.88	7.73	134.00***	5.27 [4.32, 6.34]
Self-reported attraction to early – mid pubescents	7.57	2.77	1.49	1.28	22.38*** (134.04)	5.56	6.58	1443.50***	3.27 [2.67, 3.81]
Self-reported attraction to adults	4.17	2.57	9.64	0.91	-22.18*** (125.33)	-5.95	-5.00	528.00***	-3.43 [-3.91, -2.89]
Age	34.46	13.44	35.67	11.45	-0.89 (379)	-3.85	1.53	13562.50	-0.10 [-0.34, 0.14]
PNCSE-A	1.59	1.22	1.40 [b]	0.95	1.65 (378)	-0.05	0.44	14479.50	0.18 [-0.06, 0.44]
PNCSE-P	2.71	1.62	1.80 [b]	1.24	5.35*** (172.28)	0.57	1.23	10307.00***	0.67 [0.41, 0.92]
CTQ Sexual Abuse	1.57	1.08	1.26 [b]	0.65	2.79** (149.83)	0.10	0.52	12474.00***	0.38 [0.12, 0.63]
CTQ Emotional Neglect	2.24	0.94	2.15 [b]	0.91	0.91 (378)	-0.11	0.35	14358.50	0.10 [-0.12, 0.32]
CTQ Emotional Abuse	1.89 [a]	0.85	1.83 [b]	0.91	0.62 (377)	-0.14	0.26	13735.50	0.07 [-0.15, 0.27]
CTQ Physical Neglect	1.45	0.53	1.59 [b]	0.67	-2.14* (267.77)	-0.27	0.003	13793.50	-0.22 [-0.40, -0.02]
CTQ Physical Abuse	1.37	0.61	1.53 [b]	0.73	-2.00* (378)	-0.29	-0.01	13109.50*	-0.22 [-0.42, -0.02] [d]

Note. Participants with an equally strong attraction to adults and prepubescents or early – mid pubescent were excluded from the analyses. PNCSE-A = Perceived Non-coercive Childhood Sexual Experiences with Adults. PNCSE-P = Perceived Non-coercive Childhood Sexual Experiences with Peers. CTQ = Childhood Trauma Questionnaire.

$^{*}p < .05$, $^{**}p < .01$, $^{***}p < .001$

[a] $n = 113$

[b] $n = 266$

[c] based on 1,000 bootstrap samples, bias corrected and accelerated (BCa) intervals

[d] bootstrap confidence intervals for Cohen's *d* calculated with the R package bootSE based on 2000 bootstrap resamples. As these calculations are based on different bootstrap samples than the BCa intervals of the mean difference, it is possible that statistical significance changes.

Table S6. Intercorrelations of study variables (Study 2, N = 379-381).

Variables	PNCSE_A	PNCSE_P	CTQ SA	CTQ EN	CTQ EA	CTQ PN	CTQ PA	Age
PNCSE-A	-							-
PNCSE-P	$.49^{***}$	-						-
CTQ Sexual Abuse (SA)	$.37^{***}$	$.33^{**}$	-					-
CTQ Emotional Neglect (EN)	$.11^{*}$	$.13^{**}$	$.29^{***}$	-				-
CTQ Emotional Abuse (EA)	$.14^{**}$	$.16^{**}$	$.42^{***}$	$.63^{***}$	-			-
CTQ Physical Neglect (PN)	$.29^{***}$	$.19^{***}$	$.38^{***}$	$.60^{***}$	$.55^{***}$	-		-
CTQ Physical Abuse (PA)	$.21^{***}$	$.10^{*}$	$.37^{***}$	$.47^{***}$	$.67^{***}$	$.53^{***}$	-	-
Age	.00	$.12^{*}$	.09	-.03	.03	-.07	.04	
RSPC	.07	$.28^{***}$	$.18^{***}$	.06	.04	-.09	$-.10^{*}$	-.03

$^{*}p < .05$, $^{**}p < .01$, $^{***}p < .001$

PNCSE-A = Perceived Non-coercive Childhood Sexual Experiences with Adults. PNCSE-P = Perceived Non-coercive Childhood Sexual Experiences with Peers. CTQ = Childhood Trauma Questionnaire. RSPC = Relative Sexual Preference for Children (i.e., self-reported maximum sexual attraction to prepubescent and early/mid pubescent children - self-reported maximum sexual attraction to mature adults).

Supplemental Material C.

Table S7. Distribution of item responses for perceived non-coercive childhood sexual experiences with adults and perceived non-coercive childhood sexual experiences with peers for self-reported pedohebephilic and teleiophilic group.

Study	Scale name	Items	Group	Distribution										
				never true		rarely true		sometimes true		often true		very often true		Total *N*
				n	%	*n*	%	*n*	%	*n*	%	*n*	%	
Study 1	PNCSE_A	... I had positive sexual experiences with an adult	Self-reported Pedohebephilia	81	81.0	4	4.0	7	7.0	4	4.0	4	4.0	100
			Self-reported Teleiophilia	85	95.5	0	0.0	4	4.5	0	0.0	0	0.0	89
		... I engaged in sexual acts with an adult without having been coerced or forced to do so.	Self-reported Pedohebephilia	84	83.2	3	3.0	7	6.9	3	3.0	4	4.0	101
			Self-reported Teleiophilia	86	96.6	0	0.0	3	3.4	0	0.0	0	0.0	89
Study 2	PNCSE_A	... I had positive sexual experiences with an adult	Self-reported Pedohebephilia	223	80.2	12	4.3	17	6.1	9	3.2	17	6.1	278
			Self-reported Teleiophilia	258	81.6	20	6.3	9	2.8	13	4.1	16	5.1	316
		... I engaged in sexual acts with an adult without having been coerced or forced to do so.	Self-reported Pedohebephilia	227	81.7	10	3.6	14	5.0	9	3.2	18	6.5	278
			Self-reported Teleiophilia	265	83.9	16	5.1	10	3.2	11	3.5	14	4.4	316
	PNCSE_P	... I had positive sexual experiences with same-aged peers (not older or younger than 2 years compared to myself).	Self-reported Pedohebephilia	111	39.9	34	12.2	42	15.1	32	11.5	59	21.2	278
			Self-reported Teleiophilia	196	62.0	29	9.2	45	14.2	26	8.2	20	6.3	316
		... I engaged in sexual acts with same-aged peers (not older or younger than 2 years compared to myself) without having been coerced or forced to do so.	Self-reported Pedohebephilia	116	41.7	31	11.2	39	14.0	32	11.5	60	21.6	278
			Self-reported Teleiophilia	199	63.0	29	9.2	40	12.7	24	7.6	24	7.6	316

Note. PNCSE_A = Perceived Non-coercive Childhood Sexual Experiences with Adults, PNCSE_P = Perceived Non-coercive Childhood Sexual Experiences with Peers; Instruction: "The following questions ask about some of your experiences growing up as a child and a teenager, that is, before the age of 14. Although these questions are of a personal nature, please try to answer as honestly as you can. For each question, circle the response that best describes how you feel. When I was 13 years old or younger...."
